# Initial Evaluation of AF78: a Rationally Designed Fluorine-18-Labelled PET Radiotracer Targeting Norepinephrine Transporter

**DOI:** 10.1007/s11307-019-01407-5

**Published:** 2019-07-22

**Authors:** Xinyu Chen, Alexander Fritz, Rudolf A. Werner, Naoko Nose, Yusuke Yagi, Hiroyuki Kimura, Steven P. Rowe, Kazuhiro Koshino, Michael Decker, Takahiro Higuchi

**Affiliations:** 1 Department of Nuclear Medicine, University Hospital of Würzburg, Oberdürrbacher Strasse 6, 97080 Würzburg, Germany; 2 Comprehensive Heart Failure Center, University Hospital of Würzburg, Würzburg, Germany; 3 Institute of Pharmacy and Food Chemistry, University of Würzburg, Am Hubland, 97074 Würzburg, Germany; 4 Graduate School of Medicine, Dentistry and Pharmaceutical Sciences, Okayama University, Okayama, Japan; 5 Department of Analytical and Bioinorganic Chemistry, Division of Analytical and Physical Sciences, Kyoto Pharmaceutical University, Kyoto, Japan; 6 Division of Nuclear Medicine and Molecular Imaging, Russel H. Morgan Department of Radiology and Radiological Science, Johns Hopkins University School of Medicine, Baltimore, MD USA; 7 Department of Systems and Informatics, Hokkaido Information University, Ebetsu, Hokkaido Japan

**Keywords:** Norepinephrine transporter, Positron emission tomography, Phenethylguanidine, [^18^F]AF78

## Abstract

**Purpose:**

Taking full advantage of positron emission tomography (PET) technology, fluorine-18-labelled radiotracers targeting norepinephrine transporter (NET) have potential applications in the diagnosis and assessment of cardiac sympathetic nerve conditions as well as the delineation of neuroendocrine tumours. However, to date, none have been used clinically. Drawbacks of currently reported radiotracers include suboptimal kinetics and challenging radiolabelling procedures.

**Procedures:**

We developed a novel fluorine-18-labelled radiotracer targeting NET, AF78, with efficient one-step radiolabelling based on the phenethylguanidine structure. Radiosynthesis of AF78 was undertaken, followed by validation in cell uptake studies, autoradiography, and *in vivo* imaging in rats.

**Results:**

[^18^F]AF78 was successfully synthesized with 27.9 ± 3.1 % radiochemical yield, > 97 % radiochemical purity and > 53.8 GBq/mmol molar activity. Cell uptake studies demonstrated essentially identical affinity for NET as norepinephrine and *meta*-iodobenzylgaunidine. Both *ex vivo* autoradiography and *in vivo* imaging in rats showed homogeneous and specific cardiac uptake.

**Conclusions:**

The new PET radiotracer [^18^F]AF78 demonstrated high affinity for NET and favourable biodistribution in rats. A structure-activity relationship between radiotracer structures and affinity for NET was revealed, which may serve as the basis for the further design of NET targeting radiotracers with favourable features.

**Electronic supplementary material:**

The online version of this article (10.1007/s11307-019-01407-5) contains supplementary material, which is available to authorized users.

## Introduction

In recent years, the development of positron emission tomography (PET) technology has increasingly been explored for cardiac and other applications due to its higher spatial and temporal resolution compared to single photon emission computed tomography (SPECT) [1]. This allows for a broader range of potential functional and kinetic analyses [2]. Fluorine-18-labelled radiotracers (physical half-life, 110 min) maximize the potential applicability of PET compared to carbon-11-labelled radiotracers (physical half-life, 20 min). As a result of the longer half-life and other intrinsic advantages, fluorine-18-labelled agents (1) obviate the need for on-site cyclotrons and allow central distribution of radiotracers; (2) facilitate performing a large number of scans each day; and (3) allow flexible radiotracer design and possible improvement of stability against metabolism [3].

**Fig. 1:**
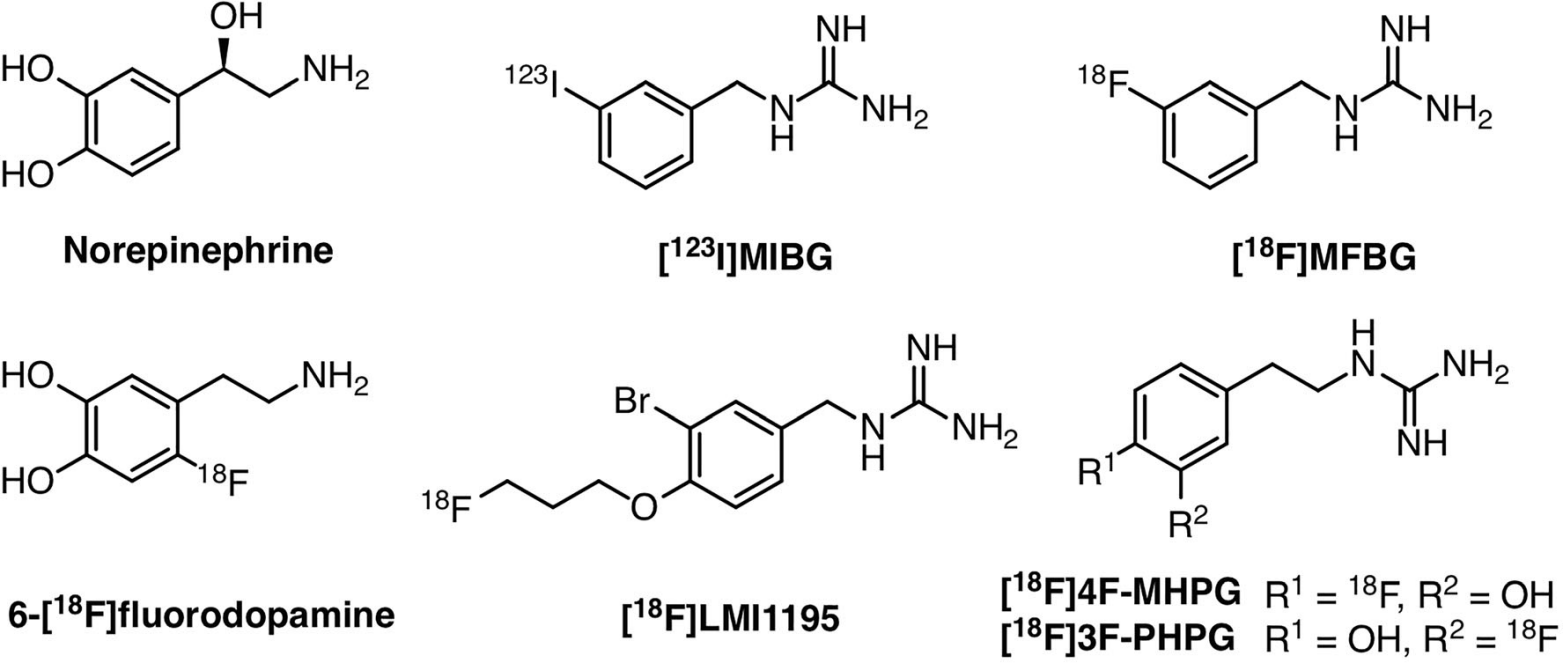
Chemical structures of the neurotransmitter norepinephrine and selected radiotracers derived from it.

To date, a handful of fluorine-18-labelled PET radiotracers have been reported that target norepinephrine transporter (NET). The interest in NET is because it is not only a major diagnostic focus in cardiovascular diseases [4–6] but also a therapeutic target in neuroendocrine tumours [7]. Typical examples include (1) 6-[^18^F]fluorodopamine, which was first reported more than 20 years ago [8, 9]; (2) [^18^F]*meta*-fluorobenzylguanidine ([^18^F]MFBG), which is the fluoride analogue of the only clinically used NET radiotracer [^123^I]*meta*-iodobenzylguanidine ([^123^I]MIBG), a single-photon emitting compound [10, 11]; (3) 1-(3-bromo-4-(3-[^18^F]fluoropropoxy)benzyl)guanidine ([^18^F]LMI1195), which shares comparable *in vitro* and *in vivo* characteristics as [^123^I]MIBG due to a common benzylguanidine core structure [12–14]; and (4) 4-[^18^F]fluoro-3-hydroxyphenethylguanidine ([^18^F]4F-MHPG) and its isomer 3-[^18^F]fluoro-4-hydroxyphenethylguanidine ([^18^F]3F-PHPG), which have shown unique slow uptake kinetics due to their phenethylguanidine core structures [15, 16] (Fig. 1). Among these radiotracers, [^18^F]LMI1195 [17] and [^18^F]4F-MHPG/3F-PHPG [18, 19] have been used mainly for cardiac sympathetic innervation imaging and have shown acceptable safety and favourable properties in clinical phase 1 trials.

**Fig. 2:**
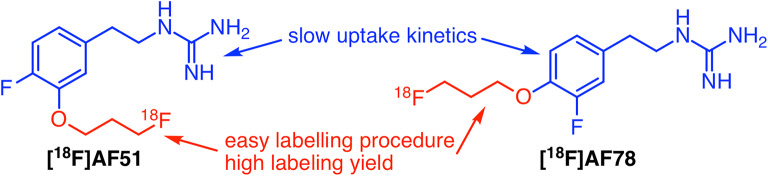
The strategy to develop new fluorine-18-labelled PET tracers targeting sympathetic nerve system along with proposed structures of target tracers.

As of now, there is no PET radiotracer that has been approved by a regulatory body for clinical use, because all the fluorine-18-labelled radiotracers mentioned above still hold some drawbacks: First, the high cardiac extraction of easily labelling [^18^F]LMI1195 is a limitation for kinetic analysis, since its uptake is consequently blood flow dependent. Second, [^18^F]4F-MHPG/3F-PHPG shows slow uptake that enables prospective quantitative analysis, but poor radiolabelling yields due to the nature of radiofluorination on electron-rich benzene ring hinders their broader application. In order to provide clinicians more choices and to investigate the relationships between the structures of radiotracers and affinity for NET, we propose two new fluorine-18-labelled compounds, AF51 and AF78. They also represent phenethylguanidine core structure of [^18^F]4F-MHPG and [^18^F]3F-PHPG, respectively, yet with 3-fluoropropoxy substitution (Fig. 2)—a strategy to develop [^18^F]LMI1195 from [^123^I]MIBG. Thus, a new series of NET targeting fluorine-18-labelled PET radiotracers with advantageous characteristics are designed: They bear the pharmacophore that is required for slow uptake kinetics along with simple radiofluorination moiety that would be more suitable for potential clinical application with higher radiochemical yield. It is noteworthy that such compounds are not a simple combination of chemical structures but might also reveal the pattern of substitution tolerability and provide new insights into radiotracer design.

## Materials and Methods

### General

Solvents were dried according to published methods and freshly distilled before use. All other reagents were commercially available compounds and were used without further purification unless noted otherwise. All reactions were carried out under a nitrogen atmosphere. H-1 and C-13 NMR spectral data were obtained from a Bruker Avance II 400 MHz NMR scanner. H-1 and C-13 NMR chemical shifts (*δ*) are reported in parts per million (ppm) relative to internal standard TMS, and coupling constants (*J*) are in hertz. Thin layer chromatography was performed on silica gel 60 with a 254-nm fluorescent indicator. UV light or iodine vapour were used for detection. Purity was evaluated on an LCMS system by Shimadzu Products, containing a LC20AB liquid chromatograph, an SPD-20A UV/Vis detector and a DGU-20A3R degassing unit. Stationary phase was a Synergi 4 μm fusion-RP (150 × 4.6 mm) column (Phenomenex, Aschaffenburg, Germany). Mass spectra were obtained by a Shimadzu LCMS-2020 (confirming purity ≥ 95 %). For column chromatography, silica gel 60, 230–400 mesh by Merck was used. For preparative thin layer chromatography, silica gel 60 PF_254_ by Merck was used.

### Radiochemistry

[^18^F]Fluoride trapped on a Sep-Pak QMA cartridge was first washed with distilled water (3 ml) to remove [^18^O]water. [^18^F]Fluoride was then eluted from the cartridge with K_2_CO_3_ solution (0.3 ml, 23 μmol/ml) into a vial that contained Kryptofix_222_ (22.5 mg, 59.7 μmol). Acetonitrile (4 × 0.5 ml) was added to the vial, and the solution was dried azeotropically under argon at 120 °C. Labelling was carried out by adding the solution of the precursor (4.8 mg) in dry acetonitrile (0.3 ml), followed by heating and stirring at 110 °C for 10 min. Hydrochloric acid (6 N, 0.3 ml) was added to the reaction mixture, and the reaction continued for 20 min at 100 °C. The mixture was cooled, diluted with 1 ml of a mixture solution of water and acetonitrile (1:1), and applied to the semi-preparative HPLC for purification. HPLC conditions were as follows: column 9.4 × 250 mm 5-μm ZORBAX Eclipse XDB-C18 (P.N. 990967-202). Mobile phase: Phase A: Millipore water with 0.1 % formic acid; Phase B: Methanol with 0.1 % formic acid. Condition: 0–20 min, 30–60 % B, 20–22 min 60–100 % B, 22–50 min 100 % B; Flow rate 3 ml/min. Condition of autoradiography: TLC with normal phase silica gel 60. Solvent system: 3 ml methanol + 20 μl formic acid, Rf.: 0.6.

### Competitive Cell Uptake Studies

SK-N-SH cells expressing NET were cultivated according to the instructions from the supplier (Sigma-Aldrich Chemie GmbH, Munich, Germany). The cells were transferred to a 12 well plate and incubated in 1 ml Eagle’s minimum essential medium (EMEM) while reaching 2 × 10^5^ cells/well density on the day for testing. The medium was removed, and the cells were washed with 1 ml EMEM. A solution of [^131^I]MIBG (Freiburg im Breigau, Germany) or [^18^F]AF78 (3.7 kBq) in EMEM (700 μl) was added to each well together with the testing compounds (norepinephrine, 4F-MHPG and cold reference AF78, each to a final concentration 100 nM, 1 μM, 10 μM and 100 μM, respectively) with or without the inhibitor desipramine (10 μM). The plate was incubated for 60 min at 37 °C. The cells were washed with ice-cold PBS buffer (2 × 1 ml) in order to remove unbound radiotracer. NaOH solution (0.1 N, 500 μl) was added to each well followed by the collection of cell pellet and measurement in a γ-counter (FH412, Frieseke & Höpfner, Erlangen, Germany) using differential energy windows (± 20 %) for fluorine-18 and iodine-131.

### *Ex vivo* Autoradiography and Tissue Counting Studies

Standard protocols and data analysis methods for non-invasive PET imaging of small animals have been established in our group [20]. Healthy male Wistar rats (Japan SLC, Inc., Shizuoka, Japan) each weighing 200–250 g were utilized. The rats were anaesthetized with 2 % isoflurane. Ten to 20 MBq of [^18^F]AF78 were administered *via* tail vein injection. Ten minutes after radiotracer administration, the animals were euthanized. Hearts, livers and blood were obtained for *ex vivo* analysis with autoradiography (Typhoon FLA 7000) and tissue counts with a γ-counter (1480 WIZARD™ 3″). Following weight and decay correction of tissue counts, the heart-to-blood (H/B) and heart-to-liver (H/L) count ratios were calculated. For autoradiography, the rats under anaesthesia were first injected *via* tail vein either with or without the NET blocker phenoxybenzamine (50 mg/kg). After 10 min, the radiotracer (10–20 MBq) was administered. The hearts were harvested 10 min later, frozen, and cut into 20 μm short axis slices using a cryostat (Cryotome FSE, Thermo Fisher Scientific), which were then immediately exposed to the autoradiography plates (Fuji SR-type image plate, Fujifilm Corporation, Tokyo, Japan) for 18 h to obtain the radiotracer distribution. The images were obtained using a digital autoradiographic system (Typhoon FLA 7000).

### Cardiac PET Imaging in Animals

Anaesthesia was induced in male Wistar rats by using 5 % isofluorane and was maintained during the whole experiment with 2 % isoflurane. All scans were obtained using a dedicated small-animal PET system (microPET FOCUS 120, SIEMENS, Munich, Germany). The PET imaging protocol was designed to assess the systemic and myocardial radiotracer distribution of [^18^F]AF78. Shortly before the injection of [^18^F]AF78 (10–20 MBq) *via* the tail vein, a 10 min dynamic PET scan was initiated with the acquisition in list-mode format. In order to evaluate the cardiac uptake mechanism of the radiotracer, rats were pretreated with phenoxybenzamine (50 mg/kg intravenously, Sigma-Aldrich, Tokyo, Japan) 10 min before radiotracer injection (10–20 MBq). The data were sorted into 3-dimensional sinograms, which were then reconstructed with a Fourier transform to produce dynamic images using a 2-dimensional ordered-subset expectation maximization (OSEM) algorithm. All images were corrected for fluorine-18 decay, random and dead time; correction for attenuation was not performed [12]. The obtained PET images were analysed with the public domain tool AMIDE imaging software (A Medical Imaging Data Examiner, version 1.01).

### Statistical Analysis

All results are displayed as mean ± SD. The two-tailed paired Student’s *t* test was used to compare differences between two dependent groups, and the two-tailed independent Student’s *t* test for differences between independent groups. A *p* value of less than 0.05 was assumed to be statistically significant. Statistical analysis was performed with StatMate III (ATMS Co., Ltd., Tokyo, Japan).

## Results

### Synthesis

For details regarding radiotracer synthesis and approaches as well as mechanism discussions, please refer to Electronic Supplementary Material (ESM). In short, in order to determine affinity to NET and to serve as references for the labelled compound, cold references (nonradioactive fluorine-19 compounds) of the target radiotracers AF51 and AF78 (Fig. 2) were first synthesized based on the reported approaches with similar structures [16, 21] (Figs. S1–S3, c.f. ESM). After the evaluation in cell uptake studies as compared to norepinephrine and MIBG, AF78 retains the same affinity as its lead compound and showed almost equal affinity to norepinephrine, whereas AF51 lost NET affinity (Fig. 4). Consequently, only the precursor of AF78 was pursued further.

**Fig. 3:**
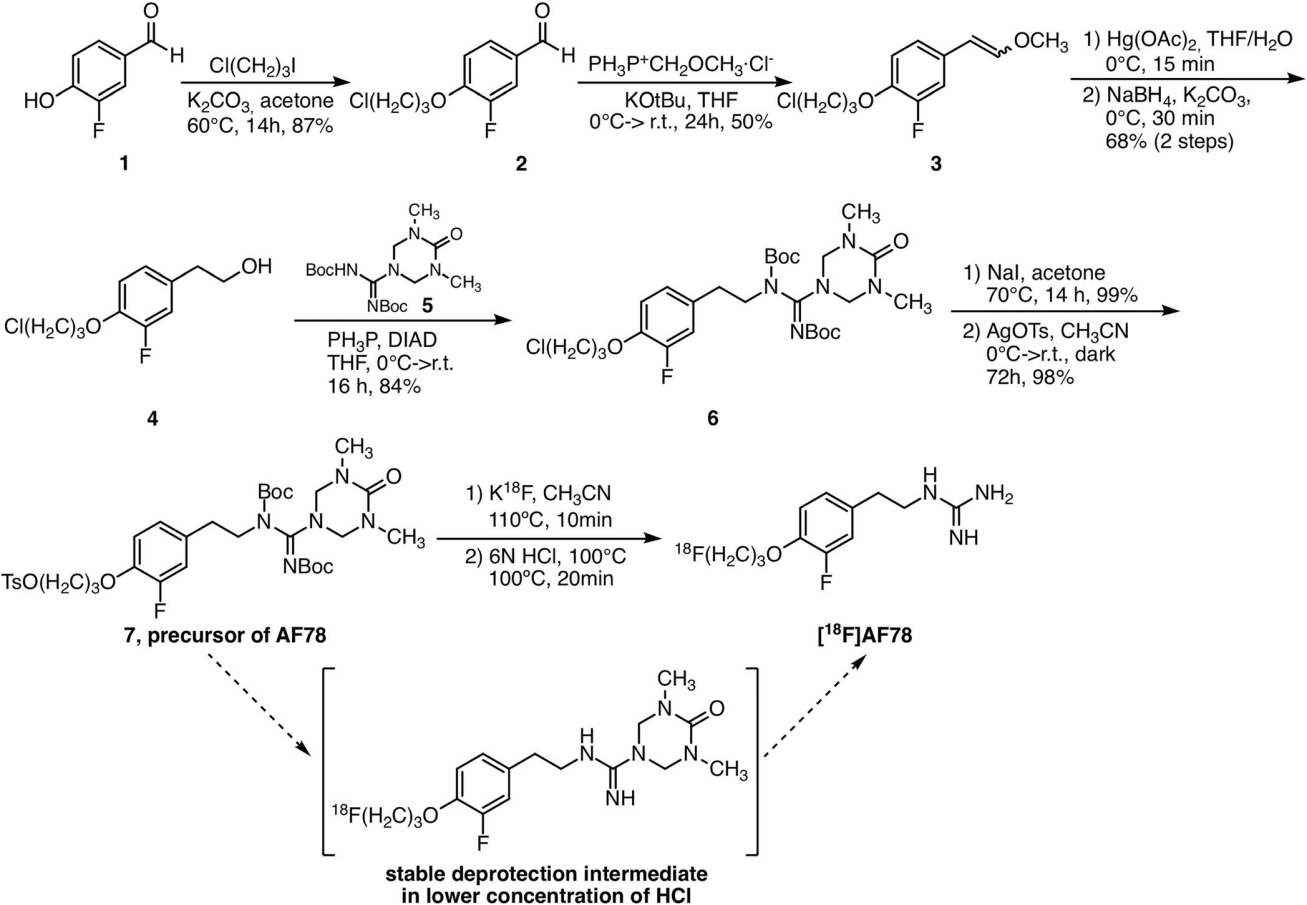
Synthetic scheme to obtain the precursor of AF78 along with the radiolabelling to generate [^18^F]AF78, which was prepared from its precursor in a one-pot, two-step labelling procedure. A deprotected intermediate was formed when using a lower concentration of HCl, which confirmed our presumption of the stability of the fully protected guanidine moiety.

As learned from the preparation of [^18^F]MFBG [22, 23], a fully protected guanidine moiety **5** (Fig. S4, c.f. ESM) was utilized to introduce this moiety to the precursor in order to improve its stability during the radiofluorination procedure and thereby facilitating a higher labelling yield. Moreover, by using a Wittig reaction followed by a one-pot oxymercuration and reduction elimination reaction [24, 25], the synthetic procedure to generate the phenethylene moiety **4** was shortened to two steps (Fig. 3) as compared to the cold references (Figs. S2 and S3 c.f. ESM). After the Mitsunobu reaction between the phenylethanol **4** and fully protected guanidine moiety **5**, tosylate was introduced as a leaving group for the radiofluorination (Fig. 3).

### Radiochemistry

Radiolabelling was performed in a one-pot, two-step reaction procedure, with the nucleophilic radiofluorination followed by deprotection of the fully protected guanidine moiety under acidic conditions. In the initial plan, the second step was done using 0.3 ml of 1 N HCl. However, after doubling the amount and concentration of acid with either longer heating time (10, 20, 30 min) or different reaction temperature (room temperature 40 °C, 70 °C), one unidentified polar peak on HPLC with significant quantity after integration did not change (Figs. S6 and S7, c.f. ESM). After several investigations on TLC-autoradiography and cold labelling attempts, the peak was eventually identified as the deprotected intermediate, *i.e.*, after the removal of both Boc groups yet still with the triazinanone structure on the guanidine (Fig. 3). This structure was more stable than expected. Therefore, 6 N HCl was used for decomposition of this moiety (Fig. S8, c.f. ESM). We consider this to be a noticeable finding, which confirmed the stability of the fully protected guanidine moiety and might be useful for labelling of other radiotracers with similar structure under relatively harsh reaction conditions. In addition to the small problem of deprotection that has been encountered, the overall radiolabelling procedure was straightforward and could be finished manually within approximately 120 min. The average radiochemical yield was 27.9 ± 3.1 % (decay-corrected based on the starting radioactivity, *n* = 5) without reaction condition optimization. The molar activity of the target radiotracer was > 53.8 GBq/mmol and the radiochemical purity > 97 % confirmed by both TLC-autoradiography (Fig. S9, c.f. ESM) and analytical HPLC (Fig. S10, c.f. ESM).

### Competitive Cell Uptake Studies

**Fig. 4:**
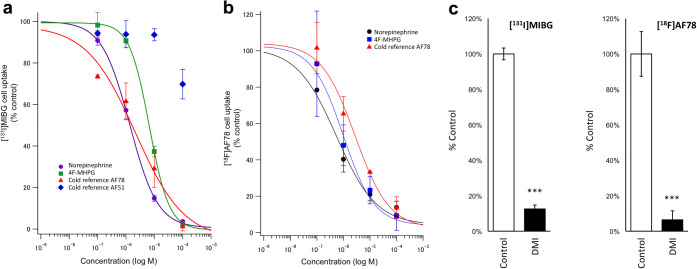
Results of the competitive cell uptake assay. **a** Dose-response curves of [^131^I]MIBG uptake in SK-N-SH cells in the presence of increasing concentrations of nonradioactive compounds; results are expressed as percent of [^131^I]MIBG uptake. Norepinephrine (violet), 4F-MHPG (green), cold reference AF51 (blue) and cold reference AF78 (red). IC_50_ values of norepinephrine, 4F-MHPG and cold reference AF78 (*n* = 3) are 1.38 ± 0.25, 6.80 ± 0.73 and 2.57 ± 1.37 μM, respectively. **b** Dose-response curves of [^18^F]AF78 uptake in SK-N-SH cells in the presence of increasing concentrations of nonradioactive compounds; results are expressed as percent of [^18^F]AF78 uptake. Norepinephrine (black), 4F-MHPG (blue) and cold reference AF78 (red) inhibited the uptake of [^18^F]AF78 at IC_50_ values of 0.60 ± 0.27, 0.85 ± 0.63 and 2.68 ± 0.83 μM, respectively. **c** Radiotracer uptake in the SK-N-SH cells in the absence and presence of NET inhibitor desipramine (DMI). [^131^I]MIBG (left, *n* = 3, 87 % inhibition compared to the control) and [^18^F]AF78 (right, *n* = 6 for control, *n* = 4 for DMI, 94 % inhibition compared to the control) uptake with and without NET inhibitor DMI. ***p < 0.001.

After successful syntheses of both cold references AF51 and AF78, they were examined in SK-N-SH cells expressing NET against the uptake of either [^131^I]MIBG [26] or [^18^F]AF78. Norepinephrine and cold MIBG were used as controls. Cold reference AF78 was able to inhibit the cell uptake of [^131^I]MIBG in a concentration-dependent manner with an IC_50_ value of 2.57 ± 1.37 μM as compared to 1.38 ± 0.25 μM and 6.80 ± 0.73 μM for norepinephrine and 4F-MHPG, respectively (Fig. 4a). In contrast, cold reference AF51 was not able to significantly inhibit the uptake of [^131^I]MIBG even at the highest concentration tested, generating only 40 % of inhibition at 100 μM. After successful radiolabelling, [^18^F]AF78 was also evaluated in SK-N-SH cells [14]. Norepinephrine (black), 4F-MHPG (blue) and cold reference AF78 (red) inhibited the uptake of [^18^F]AF78 at IC_50_ values of 0.60 ± 0.27 μM, 0.85 ± 0.63 μM, and 2.68 ± 0.83 μM, respectively (Fig. 4b). These results are in accordance with the outcomes achieved above while using [^131^I]MIBG as reference. Furthermore, the cell uptake mechanism was confirmed by the addition of the antidepressant drug desipramine (DMI), which can selectively inhibit NET-mediated transportation. Ninety-four percent of the uptake of [^18^F]AF78 was inhibited by DMI as compared to 87 % of the uptake of [^131^I]MIBG (Fig. 4c).

### Cardiac Imaging Studies in Rats

**Fig. 5:**
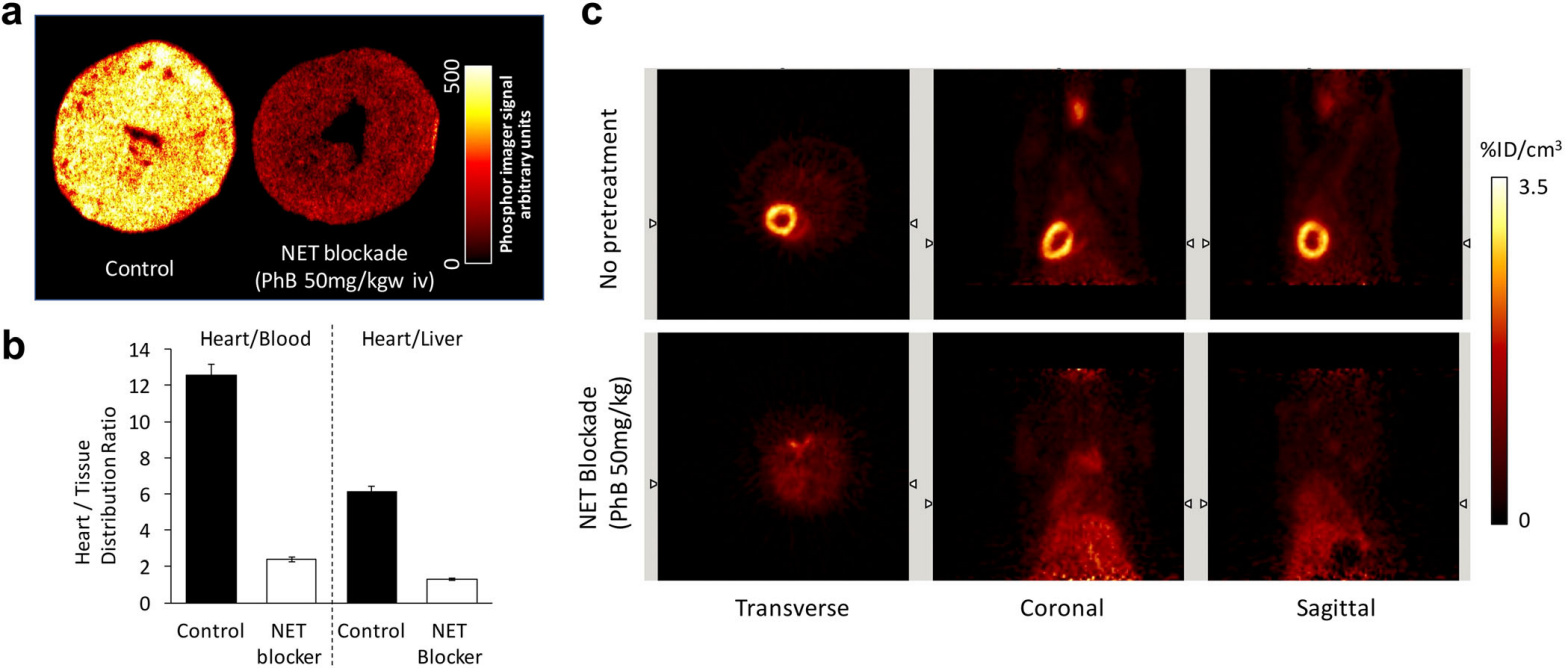
**a** Autoradiography of the left ventricular short axis slices from rats. [^18^F]AF78 demonstrated an even distribution throughout the myocardium (control), which was greatly suppressed by pretreatment with phenoxybenzamine (PhB, 50 mg/kg iv injection, NET blockade). **b** Tissue distribution ratios of [^18^F]AF78. The columns are the mean value of 2 rats. Data were determined 10 min post-radiotracer injection. *Y*-axis represents the heart/tissue distribution ratio. Both the heart-to-blood ratio and the heart-to-liver ratio decreased significantly after pretreatment with PhB (50 mg/kg iv injection, NET blockade). **c** Static PET images of cardiac uptake of [^18^F]AF78 in healthy rats, with (NET blockade) or without (no pretreatment) pretreatment with NET blocker PhB (50 mg/kg iv injection) 10 min before radiotracer injection. Homogeneous [^18^F]AF78 distribution throughout the left ventricular wall can be seen, which can be reversed by pretreatment with PhB.

In the *ex vivo* autoradiography study [20], [^18^F]AF78 showed homogenous radiotracer uptake throughout the left ventricular wall in healthy rats (Fig. 5a). A lack of delineation of the right ventricular wall might be due to its thinness and the slicing position. This accumulation was significantly suppressed by pretreatment with the NET blocker phenoxybenzamine (PhB, 50 mg/kg) 10 min before radiotracer injection (Fig. 5a).

The tissue counting study revealed that the radiotracer has selective uptake in the heart as demonstrated in both heart-to-blood (H/B) and heart-to-liver (H/L) ratios (12.54 ± 0.53 and 6.14 ± 0.35, respectively). This uptake was specifically blocked by PhB (50 mg/kg) and led to approximately 6-fold decline (≈ 81 %) in the H/B ratio and 5-fold decline (≈ 79 %) in the H/L ratio (2.41 ± 0.58 and 1.30 ± 0.54, respectively) (Fig. 5b).

A small animal PET imaging protocol was applied as described [12], from which systemic distribution of [^18^F]AF78 in the healthy rat was obtained. Homogeneous and clear radiotracer uptake throughout the left ventricular wall was observed. Though traces of delineation of the right ventricular wall were seen in transverse and coronal images, the limited discernible uptake might be due to the partial-volume effect caused by the thinness of the right ventricular wall (Fig. 5c, no pretreatment). Pretreatment with NET blocker PhB (50 mg/kg) intravenously *via* tail vein 10 min before the radiotracer injection significantly reduced the radiotracer uptake in the left ventricular myocardium counting more than 64 % decrease (Fig. 5c, NET blockade). Notably, [^18^F]AF78 showed high cardiac uptake compared to the low uptake in the adjacent organs such as blood, liver and lungs. Low bone uptake during the imaging period suggests stability at the early stage after radiotracer administration.

## Discussion

Multiple fluorine-18-labelled PET radiotracers targeting NET have been previously described. Among these reported tracers, [^18^F]LMI1195 and [^18^F]4F-MHPG/3F-PHPG have confirmed safety and tolerance in human subjects along with favourable biodistribution and kinetics suitable for NET imaging. From the chemical structural point of view, the former one possesses a benzylguanidine core structure derived from [^123^I]MIBG, while the latter ones possess phenethylguanidine core structure and show slow cardiac uptake mechanism in rat models [15, 16]. However, multiple aspects of NET radiotracer development have yet to be completely addressed. For example, one crucial question in the fluorine-18-labelled tracer design is where to introduce advantageously fluorine-18: either through nucleophilic substitution of tosylate, triflate or mesylate on an alkyl chain, such as in the case of [^18^F]LMI1195; or direct nucleophilic substitution of iodonium on the benzene ring, as illustrated by [^18^F]MFBG or [^18^F]4F-MHPG/3F-PHPG [15]. Obviously, the fluorination in the latter case is more challenging due to the electron-rich benzene ring. Therefore, the strategy to introduce fluorine as in the former case is chosen for the currently reported radiotracers. As has been demonstrated by the aforementioned results obtained from the competitive cell uptake studies of [^18^F]AF78, the introduction of 3-fluoropropoxy substitution at the *para*-position is very well tolerated. Similarly, the derivation of [^18^F]FPOIBG from [^123^I]MIBG also confirmed the feasibility of this strategy [27]. Unfortunately, the introduction of 3-fluoropropoxyl group to the *meta*-position is not tolerated by NET as we found with AF51. Nevertheless, a [^123^I]MIBG analogue *meta*-[^18^F]fluoropropyl-benzylguanidine ([^18^F]mFPBG) reported last year showing desipramine sensitive uptake-1-specific cardiac uptake in rats demonstrated a controversial finding that should raise our attention [28]. As suggested by the chemical structure of [^123^I]MIBG, the *meta*-position might only tolerate substituents up to a certain size not larger than iodine. [^18^F]LMI1195 with bromine at *meta*-position demonstrated significantly higher cardiac uptake ratio than [^123^I]MIBG [29], whereas the corresponding iodine derivative [^18^F]FPOIBG showed lower uptake in three cell line-based studies and in mice studies [27]. Similarly, the mere [^18^F]fluorine-derivative of MIBG [^18^F]FIBG, though focusing on tumour imaging, failed to represent high cardiac uptake over liver [30], which demonstrated that a mere introduction of fluorine will influence the *in vivo* character not as simple as it might appear. Moreover, although an acceptable radiochemical yield could be reached with direct benzene-ring nucleophilic fluorination, exemplified by [^18^F]MFBG (two steps, 56 min, 31 %) [23], the low radiolabelling yield of [^18^F]4F-MHPG (three steps, 150 min, 7 % and two steps, 90 min, 7 % after improvement) [16, 18] would still hinder their potentiality in clinical application. [^18^F]FPOIBG (two steps, 105 min, 5.2 %) with 3-fluoropropoxy moiety did not give higher radiochemical yield without radiolabelling procedure optimization [27], while the radiochemical yield of [^18^F]LMI1195 was not reported, neither in literature nor in patent, and is therefore difficult to compare with. We presume that the data from [^18^F]LMI1195 should be comparable to ours because of a similar radiolabelling principle. These results should be taken into consideration in the development of other NET tracers with related structures.

Furthermore, all of these radiotracers represent important monoamine oxidase resistance for long-term stable neuronal storage due to the guanidine moiety. Nevertheless, guanidine is a complex structure during the radiofluorination procedure, as any non-protected part of the moiety will interfere with the necessary nucleophilic process. One solution that has been used for the radiolabelling of [^18^F]4F-MHPG/3F-PHPG is the tetrakis-Boc protection group [18], while others chose a triazinanone structure to fully protect it as in the case of [^18^F]MFBG [22, 23]. In our study, the triazinanone structure that has been used to fully protect the guanidine moiety has demonstrated its stability during the radiolabelling procedure since it fully decomposed only after the treatment in strong acidic conditions for a long period of time. Such a protection strategy may be useful in future synthetic efforts, where a guanidine moiety needs to be introduced first but followed by harsh reaction conditions that it still remains completely protected. Moreover, in multiple-step radiolabelling procedures, the triazinanone structure could also be used to allow for more diverse radiotracer design and/or flexible labelling procedures.

Although no metabolism studies have been performed at the current stage, it can be inferred that [^18^F]AF78 is also stable against monoamine oxidase with its phenethylguanidine structure. Additionally, due to the alkylation at the *para*-hydroxyl group, the potential metabolite from sulphate conjugation, as in the case of [^18^F]4F-MHPG, will be prevented [16]. This may lead to longer radiotracer retention. A long-term dynamic imaging using [^18^F]AF78 in other animal species is currently in progress, not only to evaluate the uptake and clearance in organs of interest but also to assess the stability of 3-fluoropropoxy substituent, because slightly increased bone activity in later frames of [^18^F]LMI1195 PET studies in rats could be observed, which suggested the presence of free fluorine-18 [12]. Besides, following the current successful strategy of derivation, further effort on replacing the substitutions on the benzene-ring will also be included in future plans in the hope of gaining radiotracers with improved *in vivo* characteristics, such as longer cardiac retention time or better background contrast.

Competitive cell uptake studies revealed a NET-specific radiotracer uptake mechanism as compared with [^123^I]MIBG and norepinephrine, which was sensitive to the uptake-1 selective blocker desipramine. Different from humans, there is also an extra-neuronal uptake mechanism, *i.e.*, uptake-2, that exists in rat hearts. Therefore, in both *ex vivo* and *in vivo* studies, the cardiac uptake of [^18^F]AF78 could only be suppressed by non-selective norepinephrine/radiotracer uptake blocker phenoxybenzamine [31], which demonstrated an extra-neuronal dominant uptake of the radiotracer. Obviously, a species difference plays an important role in such a case, because both [^123^I]MIBG and [^18^F]LMI1195 have also shown significant uptake-2 mechanism preference in small rodents [12]. Larger animal studies are at the moment in progress for a deeper understanding of its *in vivo* characteristics, especially regarding the uptake kinetics, in order to gain insight into the potential applications in humans. Species differences between small rodents and other animal species, especially humans, should be taken into consideration because of the significant variations in systemic and local norepinephrine metabolism.

Cardiac imaging and blocking protocol of the current study are based on the experience investigating [^18^F]LMI1195 [12]. Indeed, 10-min of PET imaging is too short to measure delayed kinetics of radiotracer, but it is enough to reveal the initial radiotracer uptake and clearance based on our experience from [^18^F]LMI1195 studies. Besides, the main purpose of the current study is a proof of concept to design new series of NET targeting radiotracers. By showing the specific cardiac uptake in the rat hearts with good H/B and H/L ratios, we successfully demonstrated the feasibility of the design strategy presented above.

The results of tissue biodistribution studies of [^18^F]AF78, namely H/B 12.54 ± 0.53 and H/L 6.14 ± 0.35, obtained 10 min after radiotracer administration, are comparable to [^123^I]MIBG (H/B 11.91 ± 1.60, H/L 2.42 ± 0.41) and [^18^F]LMI1195 (H/B 15.56 ± 3.61, H/L 6.21 ± 1.68) [29]. Thereby, the favourable myocardium uptake of [^18^F]AF78, with low background activity, facilitates the possibility of evaluating the inferior wall activity of the heart. Again, evaluation in different animal species, especially NET-mediated radiotracer uptake-1 dominant ones, such as rabbits, is obviously needed for further comparison with currently reported radiotracers.

Indeed, there are a few PET tracers targeting the NET that have been investigated both in different animal models (mainly rats and rabbits) and in human subjects (either healthy volunteers or patients with cardiac diseases), as has been discussed in full detail in several reviews [1, 3, 32]. Carbon-11-labelled tracer hydroxyephedrine ([^11^C]HED) along with [^18^F]LMI1195 and [^18^F]4F-MHPG/3F-PHPG have drawn most attention. The former [^11^C]HED has been investigated intensively in Japan involving a large number and varieties of patients to confirm the feasibility of [^11^C]HED in quantifying regional sympathetic denervation [33], estimating myocardial blood flow [34] and to detect cardiac denervation due to Parkinson’s disease [35]. A couple of clinical trials have also been performed in Canada and in the USA. For example, the prospective PAREPET trial enrolled 204 subjects with ischemic cardiomyopathy demonstrated a strong association between the volume of denervated myocardium assessed by [^11^C]HED and cardiac events [36]. The latter fluorine-18-labelled new generation NET tracers, exemplified by [^18^F]LMI1195 and [^18^F]4F-MHPG/3F-PHPG, have been in clinical phase 1 trial with proved safety and tolerance in humans, and reported within the last decade. Consequently, not so many clinical trials on human subjects have been performed in comparison to [^11^C]HED. However, fluorine-18-labelled radiotracers might outperform current SPECT-based [^123^I]MIBG or carbon-11-labelled radiotracers in the long run, as the several advantages emphasized in the introduction section, especially due to the longer physical half-life (110 min of fluorine-18 over 20 min of carbon-11), and better spatial and temporal resolution. [^18^F]LMI1195 is derived directly from [^123^I]MIBG and shares similar *in vitro* [14] and *in vivo* [13] characteristics, including selective uptake-1 mechanism and stable storage in presynaptic storage vesicles. Differently, [^11^C]HED represents continuous leaking and reuptake equilibrium at the nerve terminal. Furthermore, clinical phase 1 trial of [^18^F]LMI1195 has demonstrated the tolerance and safety in humans but with a higher effective dose than [^123^I]MIBG [17]. Similar to the application of [^123^I]MIBG in endocrine tumours, it shows high and specific accumulation in pheochromocytomas, which represented another potential clinical application [37]. Of note, in a recently published comparison of [^18^F]LMI1195 with [^11^C]HED, preliminary data suggests a comparable estimates of cardiac sympathetic innervation but offers more favourable kinetics for early cardiac imaging [38], which shows the way to further clinical phase 2 trial in predicting arrhythmic events (NCT03493516). In addition to specific cardiac uptake with high selectivity and long period retention as defined by the authors as “irreversible neuronal retention” [18], [^18^F]4F-MHPG/3F-PHPG have drawn much attention due to their slow uptake kinetics compared to [^123^I]MIBG and [^11^C]HED, which is useful in measuring regional cardiac sympathetic nerve density quantitatively using Patlak graphical analysis [19]. One interesting point is the different metabolism of [^18^F]4F-MHPG and [^18^F]3F-PHPG in human studies [19]. As a pair of isomers, it revealed *in vivo* structure-activity relationships as [^18^F]4F-MHPG metabolized more quickly to form a sulphate at *meta*-hydroxyl position, whereas the metabolites of [^18^F]3F-PHPG remain unknown, but neither as sulphate nor as glucoside. From chemical structural point of view, this could be used to explain the even slower neuronal accumulation of [^18^F]3F-PHPG (10 min of [^18^F]4F-MHPG *vs.* 30 min of [^18^F]3F-PHPG) and higher H/L ratio of [^18^F]4F-MHPG due to its faster liver clearance (2.4 of [^18^F]4F-MHPG *vs.* 1.2 of [^18^F]3F-PHPG). Whether *in vivo* metabolism is as important as claimed remains controversial, but [^18^F]AF78 may provide an alternative by preventing the metabolism caused by a hydroxy group while potentially retaining the similar *in vivo* character with proved easy radiolabelling procedure and higher radiochemical yield. Although more detailed radiotracer kinetics of [^18^F]AF78 need to be further determined, the current study can be considered as the first step of adapting certain advantages from both [^18^F]LMI1195 and [^18^F]4F-MHPG/3F-PHPG pair.

## Conclusions

A novel PET radiotracer, [^18^F]AF78, targeting NET with a phenethylguanidine structure has been designed and evaluated. The compound demonstrated high affinity and specific cardiac uptake. Additionally, the *para*-position substitution 3-fluoropropoxyl group provided an alternative way to introduce fluorine-18 with good radiolabelling yield and radiochemical purity. The introduction of a fully protected guanidine markedly improved stability of the precursor during the synthesis and radiolabelling procedure. Favourable H/B and H/L ratios along with NET-sensitive uptake indicate that [^18^F]AF78 might allow for highly specific sympathetic nervous system imaging in human hearts, additionally with the potential of quantitative regional sympathetic nerve density measurement independent from blood flow. Furthermore, its potential application in the theranostics of neuroendocrine tumours can also be considered because of the successful clinical application of [^123^I]/[^131^I]MIBG.

## Electronic Supplementary Material

ESM 1 (PDF 892 kb)
