# Supplementary material for: Initial Evaluation of AF78: a Rationally Designed Fluorine-18-Labelled PET Radiotracer Targeting Norepinephrine Transporter
Source: Mol Imaging Biol. 2019 Jul 22;22(3):602–11. doi: 10.1007/s11307-019-01407-5 (PMC7250802; doi:10.1007/s11307-019-01407-5)
Supplement: Supplementary file 1 — (PDF 892 kb) [file 11307_2019_1407_MOESM1_ESM.pdf]

Electronic Supplementary Material

Journal: Molecular Imaging and Biology

Initial Evaluation of AF78: A Rationally Designed Fluorine-18-labelled PET Radiotracer Targeting Norepinephrine Transporter

Xinyu Chen[1,2#], Alexander Fritz[3#], Rudolf A. Werner[1,2], Naoko Nose[4], Yusuke Yagi[5], Hiroyuki Kimura[5], Steven P. Rowe[6], Kazuhiro Koshino[7], Michael Decker[3*], Takahiro Higuchi[1,2,4*]

[1]Department of Nuclear Medicine, University Hospital of Würzburg, Würzburg, Germany

[2]Comprehensive Heart Failure Center, University Hospital of Würzburg, Würzburg, Germany

[3]Institute of Pharmacy and Food Chemistry, University of Würzburg, Würzburg, Germany

[4]Graduate School of Medicine, Dentistry and Pharmaceutical Sciences, Okayama University, Okayama, Japan

[5]Department of Analytical and Bioinorganic Chemistry, Division of Analytical and Physical Sciences, Kyoto Pharmaceutical University, Kyoto, Japan

[6]Division of Nuclear Medicine and Molecular Imaging, Russel H. Morgan Department of Radiology and Radiological Science, Johns Hopkins University School of Medicine, Baltimore, Maryland, USA

[7]National Cerebral and Cardiovascular Center, Suita, Osaka, Japan

---

[#,*]These authors contributed equally to the current article.

TABLE OF CONTENTS

Chemistry………………………………………………………………………………………………S3

Radiochemistry……………………………………………………………………………………S20

In vitro assay………………………………………………………………………………………S24

References…………………………………………………………………………………………S25

Synthetic approaches to the cold references

The synthetic scheme of the cold reference of AF51 has been examined based on the reported approach with related structures [16, 21]. The methyl group from commercially available starting material **1** was removed, followed by the introduction of the 3-fluoropropoxy substituent. Henry reaction was then performed to extend the alkyl chain. Unfortunately, during reduction with lithium aluminium hydride [S1], the fluorine atom on the propyl chain was removed as well (Fig. S1). Therefore, the cold compound had to be prepared through a longer synthetic scheme (Fig. S2). The aldehyde of compound **11** was reduced, then the hydroxyl group of alcohol **14** was replaced by bromine, which could then be substituted by cyanide. Reducing conditions using borane complex were milder, which would not remove either fluorine in compound **16**. The formation of guanidine was achieved by reacting primary amine **13** with compound **17** [16, S2]. The Boc protection groups were removed by high concentration of trifluoroacetic acid (TFA) in dichloromethane to yield the cold reference AF51. Using a similar synthetic strategy, the cold reference of target tracer AF78 was also obtained (Fig. S3).

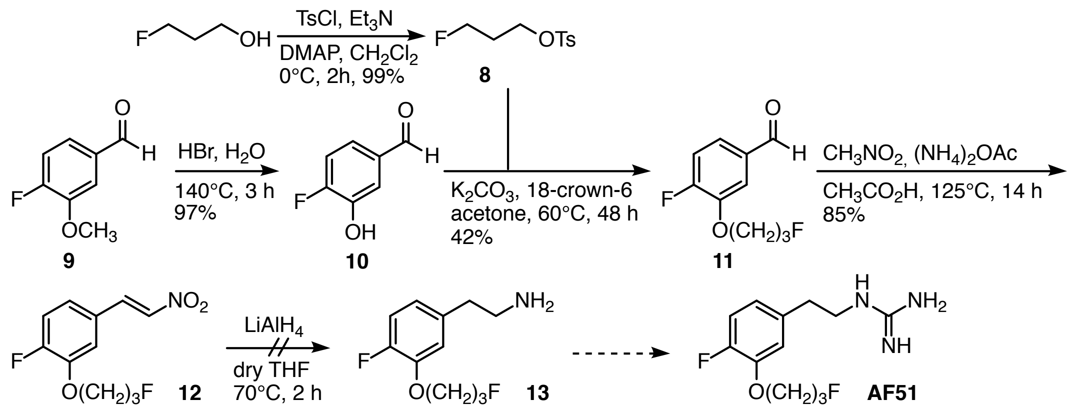

Fig. S1. Initial synthetic scheme to prepare cold reference of AF51.

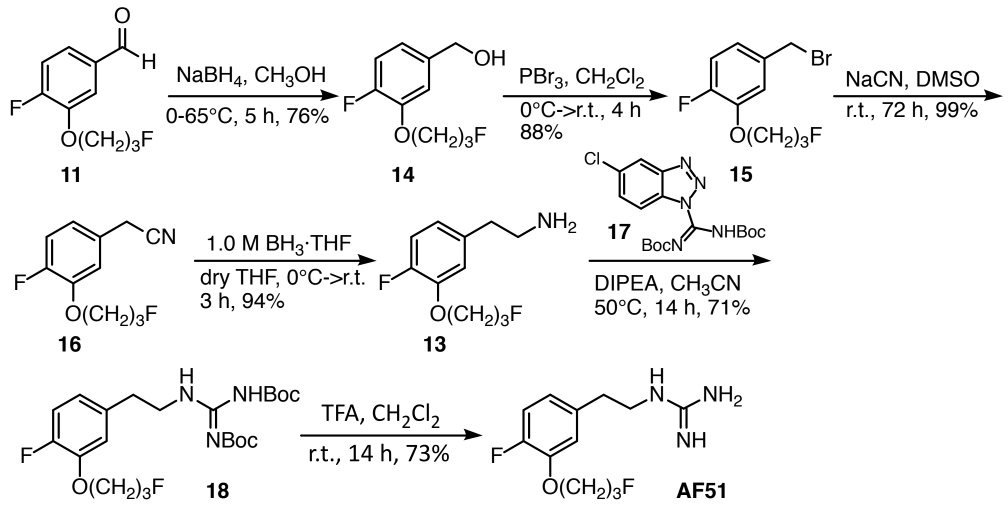

Fig. S2. Successful synthetic scheme to obtain AF51.

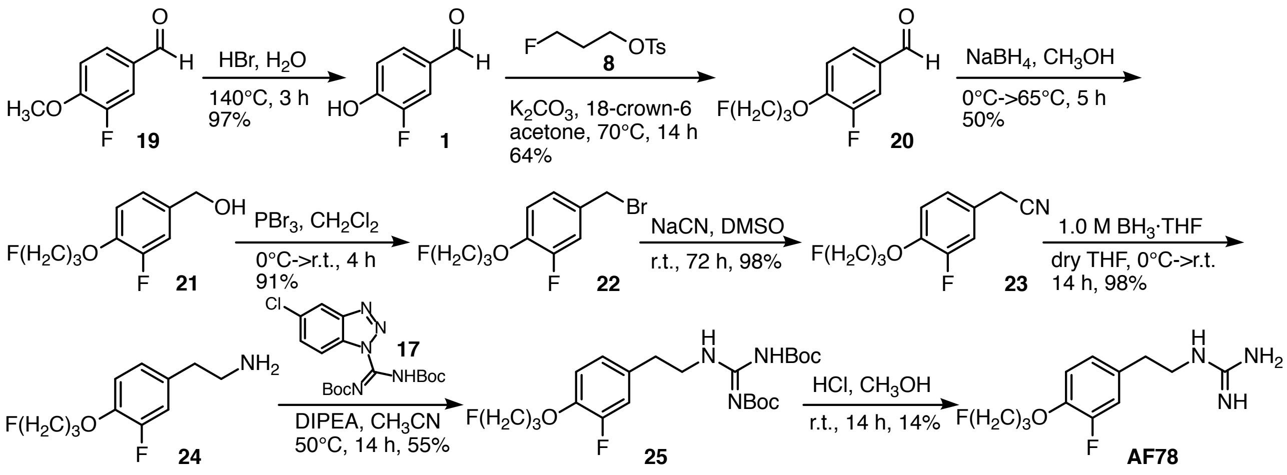

Fig. S3. Synthetic scheme to obtain AF78.

In order to synthesize the fully protected guanidine moiety **5**, a triazinanone intermediate **26** was prepared. While using *N*-benzyl triazinanone **26** to prepare **27** as reported, the high pressure (750 psi) required for hydrogenation to remove benzyl group from compound **26** [22, 23] turned out to be unfeasible for our lab condition (Fig. S4). An optimized method uses benzyl carbamate instead of benzylamine to prepare triazinanone intermediate **28**, which could use a much milder condition (room temperature stirring with ambient hydrogen pressure) to remove Cbz [S3]. While forming the fully protected guanidine moiety from **27** to **5**, reaction with pseudourea **29** proved unsuccessful. As a strong Lewis acid, the acidity

of mercury (II) chloride might lead to the decomposition of the triazinanone intermediate. Milder conditions using compound **17** were used as in the synthesis of cold references; the crucial intermediate — a fully protected guanidine moiety **5** was then obtained (Fig. S4).

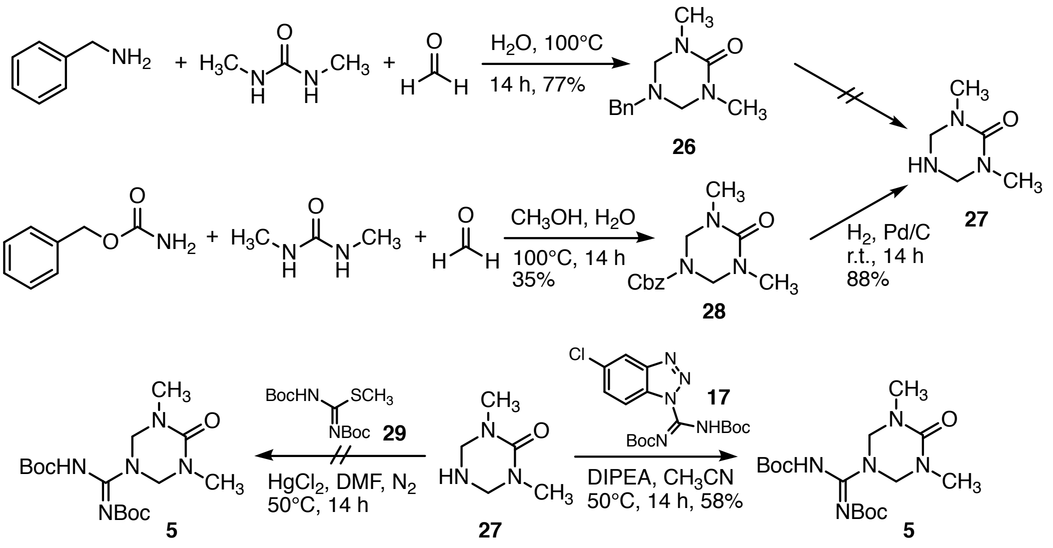

Fig. S4. Synthetic approaches to prepare fully protected guanidine moiety.

In order to prepare the precursor of [^{18}F]AF78, we were able to shorten the procedure to two steps as compared to the cold reference (Fig. 3): A Wittig reaction from benzaldehyde **2** to enol ether **3**, and a one-pot reaction to phenylethanol **4** through a phenylacetaldehyde intermediate [24]. The phenylacetaldehyde intermediate was first planned to be prepared under acidic conditions from compound **3**. But after having applied multiple demethylation conditions, such as hydrochloric acid or formic acid, no desired product could be obtained [S4, S5]. Compound **3** decomposes very fast after the addition of acid, even at low temperature (-20°C) and under anhydrous conditions, and re-formed the starting material **2**. After several attempts, a one-pot reaction using mercury (II) acetate was applied as the mercury salt could stabilize the phenylacetaldehyde intermediate, which was not isolated and could be directly reduced by addition of $NaBH_4$ in basic solution [24, 25]. As a result, the synthetic scheme was shortened with successful formation of the alcohol **4**, which was used in a Mitsunobu reaction to react with fully protected guanidine moiety **5**. The chlorine atom at the terminal of the alkyl

chain was replaced by iodine in a Finkelstein reaction, since it can subsequently form a tosylate for radiolabelling by reacting with silver tosylate in the dark (Fig. 3).

A putative mechanism of the crucial one-pot reaction is illustrated below (Fig. S5). The key in this reaction is the correct timeframe between the addition of the mercury compound and the reducing agent sodium borohydride. The vinylic double bond attacks the mercury ion, which in turn attacks one of the carbon atoms on the double bond to form the mercurinium ion intermediate. In the second step, there is a nucleophilic attack of water, which reduces the mercury ion to neutral. The methyl ether group leaves as methanol. Upon addition of sodium borohydride, the acetoxymercury group is replaced with hydrogen, resulting in the formation of the expected product and elemental mercury [S6].

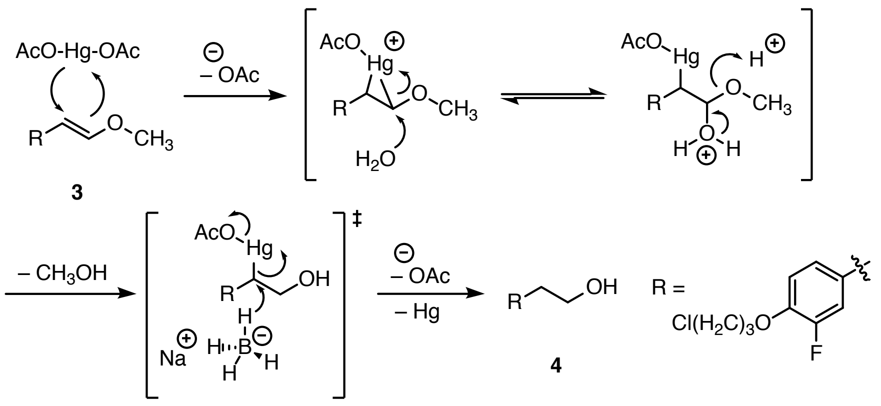

Fig. S5. Proposed mechanism of the oxymercuration and reductive elimination from compound **3** to **4**.

Experimental details

3-Fluoropropyl 4-methylbenzenesulfonate (8)

3-Fluoropropan-1-ol (3.89 g, 49.8 mmol), 4-toluenesulfonyl chloride (10.4 g, 54.8 mmol) and 4-(dimethylamino)pyridine (0.61 g, 4.89 mmol) were dissolved in dry dichloromethane. Triethylamine (7.56 g, 74.7 mmol) was added dropwise under cooling in an ice-water bath. The reaction mixture was stirred at room temperature for 2 h. The mixture was diluted with water (150 mL) and extracted with dichloromethane (4 × 100 mL). The combined organic phases were washed with citric

acid and sodium bicarbonate solutions and dried over sodium sulfate. The solvent was removed *in vacuum* to give compound **8** as a yellow liquid (11.48 g, 49.4 mmol, 99% yield). ^{1}H NMR ($CDCl_3$, 400 MHz) δ = 1.84-1.93 (m, 2 H), 2.30 (s, 3 H), 4.02 (t, 2 H), 4.26-4.40 (dt, *J* = 5.7 Hz, *J(F–H)* = 5.7 Hz, 2 H), 7.22 (d, 2 H), 7.65 (d, 2 H) ppm. ^{13}C NMR ($CDCl_3$, 101 MHz) δ = 21.25, 29.61, 29.81, 66.17, 66.22, 78.57, 80.21, 127.59, 128.79, 132.56, 144.88 ppm. ESI-MS calculated for $C_{10}H_{13}FO_3S$ m/z 233.06 $[M+H]^+$, found 233.10.

4-Fluoro-3-hydroxybenzaldehyde (10)

4-Fluoro-3-methoxybenzaldehyde (4.92 g, 31.3 mmol) was mixed with 48% hydrobromic acid (30 mL), heated to 140 °C and stirred vigorously under argon atmosphere for 3 h. The mixture was diluted with water (150 mL) and extracted with dichloromethane (2 × 100 mL). The combined organic layers were washed with brine solution and dried over sodium sulfate. The solvent was removed *in vacuum* to give compound **10** as a brown solid (4.22 g, 30.1 mmol, 97% yield). NMRs are in accordance to literature [S7]. ESI-MS calculated for $C_7H_6FO_2$ m/z 141.03 $[M+H]^+$, found 141.00.

4-Fluoro-3-(3-fluoropropoxy)benzaldehyde (11)

To a solution of compound **10** (0.94 g, 6.71 mmol), potassium carbonate (1.48 g, 10,7 mmol) and 18-crown-6 (280 mg, 1.70 mmol) in acetone, compound **8** (1.87 g, 8.05 mmol) was added and the reaction mixture was stirred at 60 °C for 48 h. The solvent was evaporated and the residue was diluted with water (100 mL) and extracted with ethyl acetate (3 × 50 ml). The combined organic phases were washed with brine and dried over sodium sulfate. The solvent was removed *in vacuum* and the crude product was purified by column chromatography (5:1 petroleum ether:ethyl acetate). The solvent was removed *in vacuum* to give compound **11** as a pale yellow liquid (0.56 g, 2.82 mmol, 42% yield). ^{1}H NMR ($CDCl_3$, 400 MHz) δ = 2.18–2.28 (m, 2H), 4.21-4.25 (t, 2H), 4.59-4.74 (m, 2 H), 7.20-7.26 (m, 1H), 7.43-7.46 (m, 1H), 7.50-7.52 (d, 1H), 9.90 (s, 1H) ppm. ^{13}C NMR ($CDCl_3$, 101 MHz) δ = 30.23, 30.43, 65.08, 65.13, 79.59-81.23 (*J(C–F)* = 165.0 Hz), 113.32, 113.36, 116.66, 116.85, 125.42, 125.51, 133.37, 133.41, 147.92, 148.03, 155.46-158.02 (*J(C–F)* = 257.7 Hz), 190.70 ppm. ESI-MS calculated for $C_{10}H_{10}F_2O_2$ m/z 201.07 $[M+H]^+$, found 201.00.

(*E*)-1-Fluoro-2-(3-fluoropropoxy)-4-(2-nitrovinyl)benzene (12)

Compound **11** (0.53 g, 2.65 mmol), ammonium acetate (0.61 g, 79.6 mmol) and nitromethane (0.97 g, 15.9 mmol) were dissolved in acetic acid and stirred at 125 °C for 14 h. The mixture was diluted with water (100 mL) and extracted with ethyl acetate (3 × 50 ml). The combined organic phases were washed with sodium hydrocarbonate, brine and dried over sodium sulfate. The solvent was removed *in vacuum* to give compound **12** as a colourless liquid (0.55 g, 22.6 mmol, 85% yield). ^{1}H NMR ($CDCl_3$, 400 MHz) δ = 1.92 (m, 0.5H), 2.26-2.36 (m, 2H), 3.81 (m, 0.5H), 4.25-4.31 (m, 2H), 4.67-4.83 (m, 2H), 7.21-7.35 (m, 2H), 7.50-7.52 (m, 1H), 7.31-7.35 (m, 1H), 7.59-8.03 (ABq, J_{AB} = 13.6 Hz, 1H) ppm. ^{13}C NMR ($CDCl_3$, 101 MHz) δ = 30.13, 30.27, 30.33, 30.47, 65.29, 65.34, 79.40, 79.56, 81.05, 81.20, 114.73, 114.75, 117.20, 117.30, 117.40, 117.50, 123.41, 123.49, 126.29, 138.28, 138.29, 147.60, 147.72, 147.83, 153.83, 154.17, 156.37 ppm. ESI-MS calculated for $C_{11}H_{11}F_2NO_3$ m/z 244.08 $[M+H]^+$, found 244.20.

(4-Fluoro-3-(3-fluoropropoxy)phenyl)methanol (14)

To a solution of compound **11** (1.22 g, 6.10 mmol) in methanol, sodium borohydride (345 mg, 9.13 mmol) was added portion wise under cooling in an ice-water bath. The reaction mixture was then heated to 65 °C and stirred for 5 h under argon atmosphere. After the reaction was completed, 5 ml of water was added and the solvent was evaporated. The crude product was washed with water and extracted with ethyl acetate (4 × 25 ml). The combined organic phases were washed with brine and dried over sodium sulfate. The solvent was removed *in vacuum* and the crude product was purified via column chromatography (2:1 petroleum ether:ethyl acetate) to give the alcohol **14** as a colourless liquid (0.93 g, 4.61 mmol, 76% yield). ^{1}H NMR ($CDCl_3$, 400 MHz) δ = 2.10-2.20 (m, 2H), 2.80-2.89 (m, 1H), 4.09-4.12 (t, *J* = 6.1 Hz, 2H), 4.53 (s, 2H), 4.55-4.58 (t, *J* = 5.7 Hz, 1H), 4.67-4.70 (t, *J* = 5.8 Hz, 1H), 6.79-6.82 (m, 1H), 6.94-7.01 (m, 2H) ppm. ^{13}C NMR ($CDCl_3$, 101 MHz) δ = 30.23, 30.43, 64.43, 64.96, 65.01, 79.76-81.39 (*J(C–F)* = 164.2 Hz), 113.66, 113.68, 115.81, 116.00, 119.46, 119.53, 127.82, 129.95, 137.36, 137.40, 146.67, 146.78, 150.75-153.19 (*J(C–F)* = 245.1 Hz) ppm. ESI-MS calculated for $C_{10}H_{12}F_2O_2$ m/z 225.07 $[M+Na]^+$, found 225.05.

4-(Bromomethyl)-1-fluoro-2-(3-fluoropropoxy)benzene (15)

To a solution of the alcohol **14** (895 mg, 4.43 mmol) in dry dichloromethane, phosphorus tribromide (599 mg, 2.21 mmol) was added dropwise under cooling in an ice-water bath. The reaction mixture was stirred at room temperature for 4 h. The mixture was diluted with water (100 mL) and extracted with dichloromethane (3 × 25 ml). The combined organic phases were washed with sodium bicarbonate, brine and dried over sodium sulfate. The solvent was removed *in vacuum* to give the bromide **15** as a yellow liquid (1.03 g, 3.90 mmol, 88% yield). Used in the next step without further purification. ESI-MS calculated for $C_{10}H_{11}BrF_2O$ m/z 185.08 $[M-Br]^+$, found 185.10.

2-(4-Fluoro-3-(3-fluoropropoxy)phenyl)acetonitrile (16)

To a solution of the bromide **15** (1.02 g, 3.85 mmol) in dimethyl sulfoxide was added sodium cyanide (189 mg, 3.85 mmol) and the reaction mixture was stirred at room temperature for 72 h. The mixture was diluted with water (50 mL), extracted with ethyl acetate (4 × 20 ml). The combined organic phases were washed with brine and dried over sodium carbonate. The solvent was removed *in vacuum* to give the nitrile **16** as a pale-yellow liquid (0.80 g, 3.81 mmol, 99% yield). ^1H NMR ($CDCl_3$, 400 MHz) δ = 2.15-2.24 (m, 2H), 3.69 (s, 2H), 4.15-4.18 (t, *J* = 6.1 Hz, 2H), 4.58-4.72 (m, 2H), 6.82-6.86 (m, 1H), 6.92-6.94 (d, *J* = 7.7 Hz, 1H), 7.03-7.08 (m, 1H) ppm. ^{13}C NMR ($CDCl_3$, 101 MHz) δ = 23.26, 30.28, 30.48, 40.91, 65.20, 65.25, 79.63-81.26 (*J(C–F)* = 164.7 Hz), 114.68, 114.70, 116.66, 116.85, 117.76, 120.72, 120.79, 126.20, 126.24, 147.28, 137.39, 151.13-153.59 (*J(C–F)* = 247.2 Hz) ppm. ESI-MS calculated for $C_{11}H_{11}F_2NO$ m/z 185.08 $[M-CN]^+$, found 185.10.

2-(4-Fluoro-3-(3-fluoropropoxy)phenyl)ethan-1-amine (13)

The nitrile **16** (0.830 g, 3.93 mmol) was dissolved in dry tetrahydrofuran and borane (1M tetrahydrofuran solution, 11.8 ml, 11.8 mmol) was added dropwise under cooling in an ice-water bath. The reaction mixture was stirred at room temperature for 3 h. After the reaction was complete, water was added and the solvent was then removed. The residue was diluted with water (50 mL) and extracted with ethyl acetate (3 × 25 ml). The combined organic phases were washed with brine and dried over sodium

sulfate. The solvent was removed *in vacuum* to give the amine **13** as viscous liquid (0.80 g, 3.70 mmol, 94% yield). Used in the next step without further purification. ESI-MS calculated for $C_{11}H_{12}F_2NO$ m/z 216.12 $[M+H]^+$, found 216.10.

tert-Butyl (_E_)-(((_tert_-butoxycarbonyl)imino)(5-chloro-1_H_-benzo[_d_][1,2,3]triazol-1-yl)methyl)carbamate (17)

1,3-Bis(tert-butoxycarbonyl)-2-methyl-2-thiopseudourea and 5-chlorobenzotriazole were dissolved in dimethylformamide and triethyl amine (0.35 g, 3.45 mol). Mercury(II) chloride ($HgCl_2$) was added and the reaction mixture was stirred at 50 °C for 24 h. The mixture was washed with sodium hydrocarbonate and water and extracted with ethyl acetate (8 × 50 ml). The combined organic layers were dried over sodium sulfate. The crude product was dissolved in dichloromethane and purified by column (5:1–1:1 petroleum ether:ethyl acetate) to give the target compound **17** as a white solid (0.74 g, 18.7 mmol. 57% yield). Used in the next step without further purification. ESI-MS calculated for $C_{17}H_{22}ClN_5O_4$ m/z 396.14 $[M+H]^+$, found 396.05.

_N_-(4-Fluoro-3-(3-fluoropropoxy)phenethyl)-_N_'-_tert_-butyloxycarbonyl-_N_''-_tert_-butyloxycarbonylguanidine (18)

To a solution of the amine **13** (370 mg, 1.72 mmol) and compound **17** (680 mg, 1.72 mmol) in acetonitrile, DIPEA (444 mg, 3.44 mmol) was added and the mixture was stirred at 50 °C for 14 h. It was then washed with sodium hydrogencarbonate, water and extracted with ethyl acetate (3 × 20 mL). The combined organic phases were washed with brine and dried over sodium sulfate. After removing the solvent *in vacuum,* the crude product was purified by column chromatography (8:1 petroleum ether:ethyl acetate) to give the title compound **18** as a white solid (562 mg, 1.23 mmol, 71% yield). ^{1}H NMR ($CDCl_3$, 400 MHz) δ = 1.49-1.52 (m, 18H), 2.16-2.25 (m, 2H), 2.82-2.85 (t, *J* = 6.9 Hz, 2H), 3.68-3.69 (m, 2H), 4.17-4.20 (t, *J* = 6.1 Hz, 2H), 4.60-4.75 (dt, *J* = 8.2, 47.0 Hz, 2H), 6.73-6.77 (m, 1H), 6.86 (m, 1H), 6.98-7.03 (m, 1H), 8.42 (s, 1H), 11.49 (s, 1H) ppm. ^{13}C NMR ($CDCl_3$, 101 MHz) δ = 28.14, 28.40, 29.83, 30.49, 35.05, 42.48, 65.05, 65.10, 79.82-81.45 (*J(C–F)* = 164.7 Hz), 115.65, 116.20, 116.38, 121.47, 121.53, 134.98, 135.02, 146.77, 146.88, 150.51, 153.32-156.18 (*J(C–F)* = 288.0 Hz) ppm. ESI-MS calculated for $C_{22}H_{33}F_2N_3O_5$ m/z 458.25 $[M+H]^+$, found 458.20.

1-(4-Fluoro-3-(3-fluoropropoxy)phenethyl)guanidine (AF51)

The protected guanidine **18** (442 mg, 0.97 mmol) was dissolved in a trifluoroacetic acid/dichloromethane (90:10) mixture and stirred at room temperature for 14 h. The solvent was removed *in vacuum*. The crude product was purified by reverse phase column chromatography (Phase A: water with 0.01% formic acid, Phase B: methanol with 0.01% formic acid. Gradient A/B 5% to 40%, 40 min) to give the target compound **AF51** as white crystals (182 mg, 0.71 mmol, 73% yield). ^{1}H NMR ($CDCl_3$, 400 MHz) δ = 2.07-2.17 (m, 2H), 2.78-2.82 (t, *J* = 7.1 Hz, 2H), 3.38-3.41 (t, *J* = 7.1 Hz, 2H), 4.11-4.14 (t, *J* = 6.1 Hz, 2H), 4.51-4.66 (dt, *J* = 47.3, 5.8 Hz, 2H), 6.75-6.79 (m, 1H), 6.95-7.00 (m, 2H) ppm. ^{13}C NMR ($CDCl_3$, 101 MHz) δ = 31.43, 31.63, 35.53, 43.62, 66.21, 66.26, 80.68-82.31 (*J(C–F)* = 163.6 Hz), 116.85, 117.04, 122.53, 122.59, 135.90, 135.94, 148.11, 148.21, 151.80-154.22 (*J(C–F)* = 243.6 Hz), 158.68 ppm. ESI-MS calculated for $C_{12}H_{17}F_2N_3O$ m/z 258.14 $[M+H]^+$, found 258.10.

3-Fluoro-4-hydroxybenzaldehyde (1)

3-Fluoro-4-methoxybenzaldehyde **19** (5.00 g, 32.5 mmol) was mixed with 48% HBr (30 mL), heated to 140 °C and stirred under argon atmosphere for 3 h. The mixture was diluted with water (150 mL) and extracted with dichloromethane (2 × 100 mL). The combined organic layers were washed with brine solution and dried over sodium sulfate. The solvent was removed *in vacuum* to give compound **1** as a brown solid (4.36 g, 30.2 mmol, 97% yield). NMRs are in accordance to literature [S8]. ESI-MS calculated for $C_7H_5FO_2$ m/z 141.03 $[M+H]^+$, found 141.00.

3-Fluoro-4-(3-fluoropropoxy)benzaldehyde (20)

To a solution of compound **1** (0.87 g, 6.21 mmol), K_2CO_3 (1.29 g, 9.32 mmol) and 18-crown-6 (264 mg, 0.93 mmol) in acetone, compound **8** (1.44 g, 6.21 mmol) was added and the reaction mixture was stirred at 70 °C for 14 h. The solvent was evaporated. The residue was diluted with water (100 mL) and extracted with ethyl acetate (3 × 50 ml). The combined organic phases were washed with brine and dried over sodium sulfate. The solvent was removed *in vacuum* and the crude product was purified by column chromatography (8:1 petroleum ether:ethyl acetate). The solvent was

removed *in vacuum* to give compound **20** as a pale yellow liquid (0.79 g, 3.94 mmol, 64% yield). ^{1}H NMR ($CDCl_3$, 400 MHz) δ = 2.20-2.29 (m, 2H), 4.24-4.27 (m, 2H), 4.60-4.75 (m, 2H), 7.07-7.11 (t, *J* = 8.1 Hz, 1H), 7.58-7.64 (m, 2H), 9.84-9.85 (m, 1H) ppm. ^{13}C NMR ($CDCl_3$, 101 MHz) δ = 30.13, 30.33, 65.05, 65.10, 79.44-81.08 (*J(C–F)* = 165.0 Hz), 113.76, 133.78, 115.64, 115.82, 127.95, 128.24, 130.00, 151.44-153.92 (*J(C–F)* = 249.8 Hz), 152.32, 152.43, 189.94, 189.96 ppm. ESI-MS calculated for $C_{10}H_{10}F_2O_2$ m/z 201.07 $[M+H]^+$, found 201.00.

(3-Fluoro-4-(3-fluoropropoxy)phenyl)methanol (21)

To a solution of compound **20** (0.71 g, 3.55 mmol) in methanol, sodium borohydride (0.20 g, 5.32 mmol) was added portion wise under cooling in an ice-water bath. The reaction mixture was then heated to 65 °C and stirred for 5 h under argon atmosphere. After the reaction, 5 ml of water was added and the solvent was evaporated. The crude product was diluted with water (50 mL) and extracted with ethyl acetate (4 × 25 ml). The combined organic phases were washed with brine and dried over sodium sulfate. The solvent was removed *in vacuum* and the crude product was purified via column chromatography (2:1 petroleum ether:ethyl acetate) to give the alcohol **21** as a colourless liquid (0.36 g, 1.79 mmol, 50% yield). ^{1}H NMR ($CDCl_3$, 400 MHz) δ = 2.02-2.21 (m, 2H), 4.13-4.16 (t, *J* = 6.1 Hz, 2H), 4.57-4.59 (m, 3H), 4.70-4.73 (t, *J* = 5.7 Hz, 1H), 6.93-7.09 (m, 3H) ppm. ^{13}C NMR ($CDCl_3$, 101 MHz) δ = 30.41, 30.60, 64.41, 65.27, 65.32, 79.84-81.48 (*J(C–F)* = 164.4 Hz), 115.10, 115.14, 115.16, 122.82, 122.86, 134.59, 134.65, 146.21, 146.32, 151.56-154.01 (*J(C–F)* = 246.4 Hz) ppm. ESI-MS calculated for $C_{10}H_{12}F_2O_2$ m/z 225.07 $[M+Na]^+$, found 225.05.

4-(Bromomethyl)-2-fluoro-1-(3-fluoropropoxy)benzene (22)

To a solution of the alcohol **21** (361 mg, 1.79 mmol) in dry dichloromethane, phosphorus tribromide (242 mg, 0.89 mmol) was added dropwise under cooling in an ice-water bath. The reaction mixture was stirred at room temperature for 4 h. The mixture was diluted with water (50 mL) and extracted with dichloromethane (3 × 25 ml). The combined organic phases were washed with sodium bicarbonate, brine and dried over sodium sulfate. The solvent was removed *in vacuum* to give the bromide **22** as a yellow liquid (433 mg, 1.63 mmol, 91% yield). Used in the next step

without further purification. ESI-MS calculated for $C_{10}H_{11}BrF_2O$ m/z 185.08 [M-Br]$^+$, found 185.10.

2-(3-Fluoro-4-(3-fluoropropoxy)phenyl)acetonitrile (23)

To a solution of the bromide **22** (433 mg, 1.69 mmol) in dimethyl sulfoxide was added sodium cyanide (87 mg, 3.85 mmol) and the reaction mixture was stirred at room temperature for 72 h. The mixture was diluted with water (40 mL), extracted with ethyl acetate (4 × 15 ml). The combined organic phases were washed with brine and dried over sodium carbonate. The solvent was removed *in vacuum* to give the nitrile **23** as a pale-yellow liquid (348 mg, 1.65 mmol, 98% yield). ^{1}H NMR ($CDCl_3$, 400 MHz) δ = 2.13-2.23 (m, 2H), 3.66 (s, 2H), 4.14 (m, 2H), 4.58-4.70 (m, 2H), 6.95-7.05 (m, 3H) ppm. ^{13}C NMR ($CDCl_3$, 101 MHz) δ = 22.79, 30.30, 30.50, 65.18, 65.23, 79.67-81.31 (*J(C–F)* = 164.6 Hz), 115.44, 115.46, 116.24, 117.69, 122.97, 123.04, 123.90, 123.94, 146.69, 146.80, 151.47-153.94 (*J(C–F)* = 248.0 Hz) ppm. ESI-MS calculated for $C_{11}H_{11}F_2NO$ m/z 185.08 [M-CN]$^+$, found 185.10.

2-(3-fluoro-4-(3-fluoropropoxy)phenyl)ethan-1-amine (24)

The nitrile **23** (340 mg, 1.61 mmol) was dissolved in dry tetrahydrofuran and borane (1M tetrahydrofuran solution, 4.83 ml, 4.83 mmol) was added dropwise under cooling in an ice-water bath. The reaction mixture was stirred at room temperature for 14 h. After the reaction, water was added and the solvent was then removed. The residue was diluted with water (40 mL) and extracted with ethyl acetate (3 × 20 ml). The combined organic phases were washed with brine and dried over sodium sulfate. The solvent was removed *in vacuum* to give the amine **24** as a viscous liquid (339 mg, 1.57 mmol, 98% yield). Directly used in the next step without further purification. ESI-MS calculated for $C_{11}H_{15}F_2NO$ m/z 216.12 [M+H]$^+$, found 216.10.

***N*-(3-Fluoro-4-(3-fluoropropoxy)phenethyl)-*N*'-*tert*-butyloxycarbonyl-*N*''-*tert*-butyloxycarbonylguanidine (25)**

To a solution of the amine **24** (340 mg, 1.58 mmol) and compound **17** (626 mg, 1.58 mmol) in acetonitrile, DIPEA (408 mg, 3.16 mmol) was added and the mixture was stirred at 50 °C for 14 h. It was then washed with sodium hydrogencarbonate, water

and extracted with ethyl acetate (3 × 20 mL). The combined organic phases were washed with brine and dried over sodium sulfate. After removing the solvent *in vacuum,* the crude product was purified by column chromatography (10:1 petroleum ether:ethyl acetate) to give the title compound **25** as a white solid (398 mg, 0.87 mmol, 55% yield). ^{1}H NMR ($CDCl_3$, 400 MHz) δ = 1.47 (s, 9H), 1.40 (s, 9H), 2.10-2.23 (m, 2H), 2.77-2.80 (t, *J* = 7.1 Hz, 2H), 3.60-3.65 (dd, *J* = 12.5, 7.0 Hz, 2H), 4.11-4.14 (t, *J* = 6.1 Hz, 2H), 4.58-4.70 (dt, 47.1, 5.7 Hz, 2H), 6.88-6.96 (m, 3H), 7.26 (s, 1H), 8.35 (s, 1H) ppm. ^{13}C NMR ($CDCl_3$, 101 MHz) δ = 28.12, 28.39, 30.47, 30.67, 34.49, 42.16, 65.28, 65.33, 77.48, 79.38, 79.81-81.45 (*J(C–F)* = 164.6 Hz), 83.20, 115.42, 115.45, 116.70, 116.89, 124.49, 124.53, 132.23, 132.29, 145.42, 145.53, 151.56-154.01 (*J(C–F)* = 246.1 Hz), 153.30, 156.26, 163.67 ppm. ESI-MS calculated for $C_{22}H_{33}F_2N_3O_5$ m/z 458.25 [M+H]$^+$, found 458.20.

1-(3-Fluoro-4-(3-fluoropropoxy)phenethyl)guanidine (AF78)

The protected guanidine **25** (210 mg, 0.46 mmol) was dissolved in a hydrochloric acid/methanol (1:2) mixture and stirred at room temperature for 14 h. The solvent was removed *in vacuum*. The residue was diluted with water (40 mL) and impurities were extracted with ethyl acetate (2 × 20 ml). The water phase was saturated with sodium chloride and extracted with dichloromethane (12 × 15 ml). The combined organic phases were dried over sodium sulfate and the solvent was removed *in vacuum.* The crude product was purified by reverse phase column chromatography (Phase A: water with 0.01% formic acid, Phase B: methanol with 0.01% formic acid. Gradient A/B 5% to 40%, 40 min) to give the target compound **AF78** as a colourless liquid (16 mg, 0.71 mmol, 14% yield). ^{1}H NMR ($CDCl_3$, 400 MHz) δ = 2.06-2.15 (dp, *J* =25.3, 6.0 Hz, 2H), 2.76-2.79 (t, *J* = 6.9 Hz, 2H), 3.35-3.39 (t, *J*, 6.9 Hz, 2H), 4.08-4.11 (t, *J* = 6.1 Hz, 2H), 4.50-4.65 (dt, *J* = 47.3, 5.8 Hz, 2H), 6.94-7.03 (m, 3H) ppm. ^{13}C NMR ($CDCl_3$, 101 MHz) δ = 31.43, 31.63, 35.01, 43.54, 66.33, 66.38, 80.69-82.31 (*J(C–F)* = 163.5 Hz), 116.51, 117.38, 117.57, 125.84, 125.88, 132.87, 132.94, 146.91, 147.01, 152.72-155.16 (*J(C–F)* = 245.0 Hz), 158.71 ppm. ESI-MS calculated for $C_{12}H_{17}F_2N_3O$ m/z 258.14 [M+H]$^+$, found 258.10.

5-Benzyl-1,3-dimethyl-1,3,5-triazinan-2-one (26)

Benzylamine (9.81 g, 91.7 mmol) was dissolved in formaldehyde (14.7 g, 183 mmol, 40% aq. solution) and heated to 100 °C under argon atmosphere. *N,N'*-dimethylurea (8.07 g, 91.7 mmol) was added and the mixture was stirred for 14 h. The mixture was washed with water and extracted with dichloromethane (2 × 200 mL). The combined organic layers were washed with brine solution and dried over sodium sulfate. The solvent was removed *in vacuum* and the crude product was purified by column chromatography (1:1 petroleum ether:ethyl acetate) to give the title compound **26** as yellow crystals (15.5 g, 70.9 mmol, 77% yield). ^{1}H NMR ($CDCl_3$, 400 MHz) δ = 2.85 (s, 6 H), 3.90 (s, 2 H), 4.16 (s, 4 H), 7.28 (m, 5 H) ppm. ^{13}C NMR ($CDCl_3$, 101 MHz) δ = 32.47, 55.36, 67.72, 127.70, 128.60, 129.11, 137.49, 156.04 ppm. ESI-MS calculated for $C_{12}H_{17}N_3O$ m/z 220.14 $[M+H]^+$, found 220.10.

Benzyl 3,5-dimethyl-4-oxo-1,3,5-triazinane-1-carboxylate (28)

Benzyl carbamate (1.00 g, 6.62 mmol) was dissolved in formaldehyde (1.18 g, 14.6 mmol, 40% aq. solution) and methanol. The mixture was heated to 100 °C under argon atmosphere, *N,N'*-dimethylurea (0.58 g, 6.62 mmol) was added and the mixture was stirred for 14 h. The solvent was removed *in vacuum* and crude product was washed with water and extracted with dichloromethane (2 × 200 mL). The combined organic layers were washed with brine solution and dried over sodium sulfate. The solvent was removed *in vacuum* and the crude product was purified by column chromatography (1:1 petroleum ether:ethyl acetate, then 25:1 dichloromethane:methanol) to give the title compound **28** as a colourless liquid (0.60 g, 2.29 mmol, 35% yield). ^{1}H NMR ($CDCl_3$, 400 MHz) δ = 2.79 (s, 6H), 4.62 (s, 4H), 5.09 (s, 2H), 7.26-7.27 (5H) ppm. ^{13}C NMR ($CDCl_3$, 101 MHz) δ = 32.66, 60.34, 68.15, 128.04, 128.40, 128.58, 135.56, 154.39, 156.07 ppm. ESI-MS calculated for $C_{13}H_{17}N_3O_3$ m/z 264.13 $[M+H]^+$, found 264.10.

1,3-Dimethyl-1,3,5-triazinan-2-one (27)

Compound **28** (603 mg, 2.29 mmol) was dissolved in methanol, palladium on activated charcoal (30 mg) was added and the reaction mixture was stirred at r.t. for 14 h under hydrogen atmosphere. The mixture was filtered and the filtrate was concentrated *in*

vacuum to give the title compound **27** as white solid (259 mg, 2.01 mmol, 88% yield). ^{1}H NMR ($CDCl_3$, 400 MHz) δ = 2.77 (s, 1H), 2.85 (s, 5H), 4.16 (s, 4H) ppm.

tert-Butyl (Z)-(((tert-butoxycarbonyl)imino)(3,5-dimethyl-4-oxo-1,3,5-triazinan-1-yl)methyl)carbamate (5)

To a solution of compound **27** (137 mg, 1.06 mmol) and compound **17** (420 mg, 1.06 mmol) in acetonitrile, DIPEA (274 mg, 2.13 mmol) was added and the mixture was stirred at 50 °C for 14 h. The solvent was removed *in vacuum*. The residue was diluted with water (50 mL) and extracted with ethyl acetate (2 × 30 ml) and dichloromethane (2 × 30 ml). The combined organic phases were washed with brine, dried over sodium sulfate and the solvent was removed *in vacuum.* The crude product was purified by column chromatography (6:1 then 2:1 petroleum ether:ethyl acetate, 1:1:0.05 petroleum ether:ethyl acetate:triethylamine) to give the target compound **5** as white crystals (226 mg, 0.61 mmol, 58% yield). ^{1}H NMR ($CDCl_3$, 400 MHz) δ = 1.47-1.48 (m, 18H), 2.01-2.02 (m, 1H), 2.90-2.91 (m, 6H), 4.69 (s, 6H) ppm. ^{13}C NMR ($CDCl_3$, 101 MHz) δ = 28.16, 33.18, 60.47, 62.31, 81.82, 154.01, 157.30 ppm. ESI-MS calculated for $C_{16}H_{29}N_5O_5$ m/z 272.22 $[M+H]^+$, found 372.20.

4-(3-Chloropropoxy)-3-fluorobenzaldehyde (2)

To a solution of compound **1** (2.50 g, 17.9 mmol) and potassium carbonate (2.71 g, 19.6 mmol) in acetone, 1-chloro-3-iodpropane (3.64 g, 17.9 mmol) was added and the reaction mixture was stirred at 60 °C for 14 h. The solvent was evaporated and the residue was diluted with water (100 mL) and extracted with ethyl acetate (2 × 50 ml). The combined organic phases were washed with brine and dried over sodium sulfate. The solvent was removed *in vacuum* and the crude product was purified by column chromatography (5:1 petroleum ether:ethyl acetate). The solvent was removed *in vacuum* to give compound **2** as a pale-yellow liquid (3.36 g, 15.6 mmol, 87% yield). ^{1}H NMR ($CDCl_3$, 400 MHz) δ = 2.23-2.32 (m, 2 H), 3.74-3.78 (m, 2 H), 4.22-4.28 (m, 2 H), 7.58-7.80 (m, 3 H), 9,85 (s, 1 H) ppm. ^{13}C NMR ($CDCl_3$, 101 MHz) δ = 31.98, 41.14, 65.69, 113.62, 113.81, 115.94, 116.15, 123.30, 123.37, 130.20, 130.30, 147.54, 148.66, 150.66-153.12 (*J(C–F)* = 254.4 Hz), 189.96 ppm. ESI-MS calculated for $C_{10}H_{10}ClFO_2$ m/z 217.04 $[M+H]^+$, found 217.10.

(*E*)-1-(3-Chloropropoxy)-2-fluoro-4-(2-methoxyvinyl)benzene (3)

(Methoxymethyl)triphenylphosphonium chloride (538 mg, 1.57 mmol) was dissolved in dry tetrahydrofuran and cooled to 0°C. Potassium *tert*-butoxide (228 mg, 2.04 mmol) was added portionwise and the mixture was stirred for 1 h. Compound **2** (200 mg, 1.00 mmol) in dry tetrahydrofuran was added dropwise under cooling and the reaction was warmed to room temperature overnight. After mixture was quenched with water, the solvent was removed *in vacuum* and the residue was diluted with water (100 mL) and extracted with ethyl acetate (2 × 50 ml). The combined organic phases were washed with brine and dried over sodium sulfate. The solvent was removed *in vacuum* and the crude product was purified by column chromatography (15:1 petroleum ether:ethyl acetate) to give the target compound **3** as a colourless oil (192 mg, 0.78 mmol, 50% yield). ^{1}H NMR ($CDCl_3$, 400 MHz) δ = 2.25 (m, 2 H), 3.37 (s, 2 H), 3.76-3.77 (m, 3 H), 4.16 (m, 2H), 5.13-5.14 (d, *J* = 6.9 Hz, 1H), 5.70-5.73 (d, *J* = 13.0 Hz, 0.5 H), 6.08-6.10 (d, *J* = 7.0 Hz, 0.5 H), 6.87-6.89 (m, 3 H) ppm. ^{13}C NMR ($CDCl_3$, 101 MHz) δ = 32.47, 41.52, 41.55, 56.70, 60.78, 66.15, 66.29, 112.69, 112.88, 115.08, 115.10, 115.84, 115.86, 116.02, 116.21, 121.13, 121.17, 147.57, 148.78, 151.91-154.35 (*J(C–F)* = 245.1 Hz), 153.80 ppm.

2-(4-(3-Chloropropoxy)-3-fluorophenyl)ethan-1-ol (4)

Compound **3** (90 mg, 0.37 mmol) was dissolved in tetrahydrofuran and a solution of mercury(II) acetate (129 mg, 0.41 mmol) in water was added under cooling in an ice/water bath. The reaction mixture was stirred for 15 mins and sodium borohydride (56 mg, 1.48 mmol) in a saturated potassium carbonate solution was added dropwise. The reaction mixture was stirred for 30 min, diluted with water and extracted with ethyl acetate (4 × 10 ml). The combined organic phases were washed with brine and dried over sodium sulfate. The solvent was removed *in vacuum* and the crude product was purified by column chromatography (4:1 petroleum ether:ethyl acetate) to give the target compound **4** as colourless oil (58.5 mg, 0.25 mmol, 68% yield). ^{1}H NMR ($CDCl_3$, 400 MHz) δ = 2.22-2.28 (m, 2 H), 2.77-2.80 (t, *J* = 6.5 Hz, 2 H), 3.75-3.83 (m, 4 H), 4.15-4.18 (t, *J* = 5.8 Hz, 2H), 6.91-6.98 (m, 3 H) ppm. ^{13}C NMR ($CDCl_3$, 101 MHz) δ

= 31.27, 38.82, 41.32, 41.43, 62.64, 65.98, 113.39, 113.46, 116.63, 116.84, 125.48, 125.56, 133.36, 133.39, 144.45, 144.85, 153.22-156.17 (*J(C–F)* = 251.5 Hz) ppm.

***tert*-Butyl (*E*)-(((*tert*-butoxycarbonyl)imino)(3,5-dimethyl-4-oxo-1,3,5-triazinan-1-yl)methyl)(4-(3-chloropropoxy)-3-fluorophenethyl)carbamate (6)**

Compound **4** (72 mg, 0.31 mmol), compound **5** (172 mg, 0.46 mmol) and triphenyl phosphine (122 mg, 0.46 mmol) were dissolved in dry tetrahydrofuran. A solution of DIAD (94 mg, 0.46 mmol) in dry tetrahydrofuran was added dropwise at 0 °C and the reaction mixture was slow warmed up to room temperature and stirred for 16 h. The mixture was diluted with water (40 mL) and extracted with diethyl ether (4 × 10 ml). The combined organic phases were washed with brine and dried over sodium sulfate. The solvent was removed *in vacuum* and the crude product was purified by column chromatography (2.5:1.5:0.5 petroleum ether:acetone:ethyl acetate) to give the target compound **6** as a colourless liquid (152 mg, 0.26 mmol, 84% yield). ^{1}H NMR ($CDCl_3$, 400 MHz) δ = 1.47 (s, 18 H), 2.22-2.25 (m, 2 H), 2.84 (m, 6 H), 3.57 (bs, 4 H), 3.74-3.77 (m, 2 H), 4.13-4.16 (m, 2 H), 4.52 (bs, 4 H), 6.89-6.95 (m, 3 H) ppm. ^{13}C NMR ($CDCl_3$, 101 MHz) δ = 28.14, 28.25, 41.43, 48.87, 60.49, 61.02, 66.02, 80.84, 83.00, 115.48, 116.55, 116.73, 124.40, 124.43, 131.50, 131.56, 145.60, 145.71, 150.98, 151.52-153.97 (*J(C–F)* = 246.8 Hz), 152.31, 156.98, 158.39 ppm. ESI-MS calculated for $C_{27}H_{41}ClFN_5O_6$ m/z 586.28, 588.28 $[M+H]^+$, found 586.25, 588.25.

***tert*-Butyl (*E*)-(((*tert-b*utoxycarbonyl)imino)(3,5-dimethyl-4-oxo-1,3,5-triazinan-1-yl)methyl)(3-fluoro-4-(3-iodopropoxy)phenethyl)carbamate (6a)**

Compound **6** (72 mg, 0.12 mmol) and sodium iodine (37 mg, 0.25 mmol) were dissolved in acetone and the reaction mixture was stirred at 70 °C for 14 h. The mixture diluted with water (40 mL) and extracted with diethyl ether (4 x 10 ml). The combined organic phases were washed with brine and dried over sodium sulfate. The solvent was removed *in vacuum* to give the target compound **6a** as a yellow liquid (83 mg, 0.12 mmol, 99% yield) and was used directly in the next step without further purification. ESI-MS calculated for $C_{27}H_{41}FIN_5O_6$ m/z 678.22 $[M+H]^+$, found 678.20.

(*E*)-3-(4-(2-(*N,N'*-bis(*tert*-Butoxycarbonyl)-3,5-dimethyl-4-oxo-1,3,5-triazinane-1-carboximidamido)ethyl)-2-fluorophenoxy)propyl 4-methylbenzenesulfonate (7)

Compound **6a** (72 mg, 0.11 mmol) was dissolved in acetonitrile and silver p-toluenesulfonate (148 mg, 0.53 mmol) in darkness at 0 °C. The reaction mixture was stirred at room temperature for 72 h. The crude product was diluted with water (40 mL) and extracted with ethyl acetate (4 × 10 ml). The combined organic phases were washed with brine and dried over sodium sulfate. The solvent was removed *in vacuum* to give the target compound **7** as a yellow liquid (75 mg, 0.10 mmol, 98% yield). ^{1}H NMR ($CDCl_3$, 400 MHz) δ = 1.48 (s, 18 H), 2.11-2.14 (m, 2 H), 2.14 (s, 3H), 2.85 (s, 6H), 3.85 (bs, 4H), 3.89-4.01 (m, 2H), 4.22-4.25 (m, 2H), 4.55 (bs, 4H), 6.77-6.93 (m, 3H), 7.26-7.28 (m, 2H), 7.75-7.77 (d, *J* = 8.3 Hz) ppm. ^{13}C NMR ($CDCl_3$, 101 MHz) δ = 28.15, 28.26, 29.05, 29.80, 33.22, 33.66, 60.50, 61.20, 64.87, 66.98, 80.93, 83.06, 115.32, 116.49, 116.67, 124.42, 127.98, 129.95, 131.55, 132.92, 144.92, 145.36, 145.47, 151.37, 152.28, 153.82, 156.95 ppm. ESI-MS calculated for $C_{34}H_{48}FN_5O_9S$ m/z 722.32 $[M+H]^+$, found 722.35.

Radiochemistry

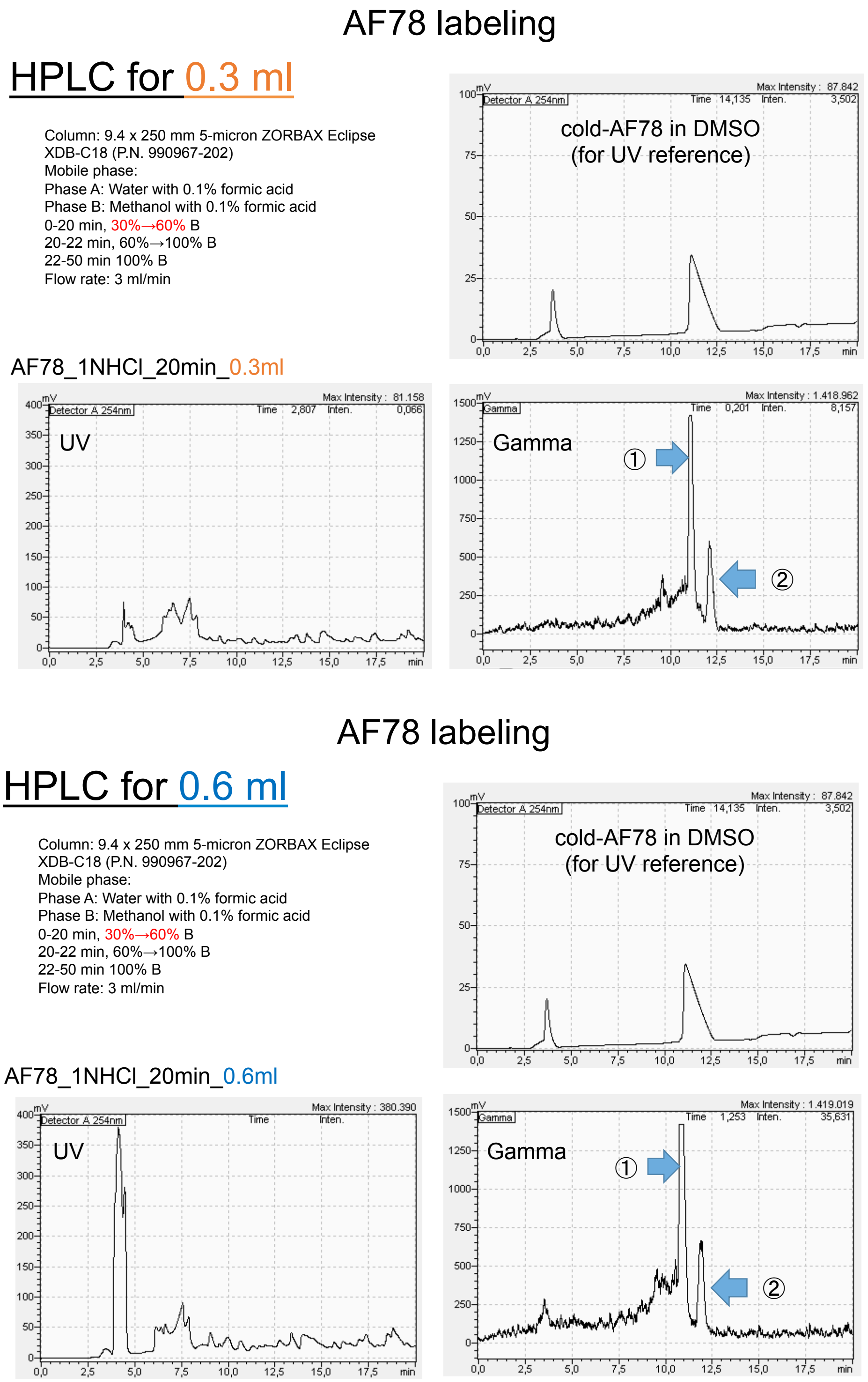

Fig. S6. HPLC with UV and gamma detector after the deprotection step in comparison with the cold reference. Conditions: above, 0.3 mL 1N HCl 100°C for 20 min; below, 0.6 mL 1N HCl 100°C for 20 min. Peak 1 is the unidentified peak that has formed, which has been confirmed still with the structure of triazanone on guanidine. Peak 2 is the target radiolabeled tracer [^{18}F]AF78.

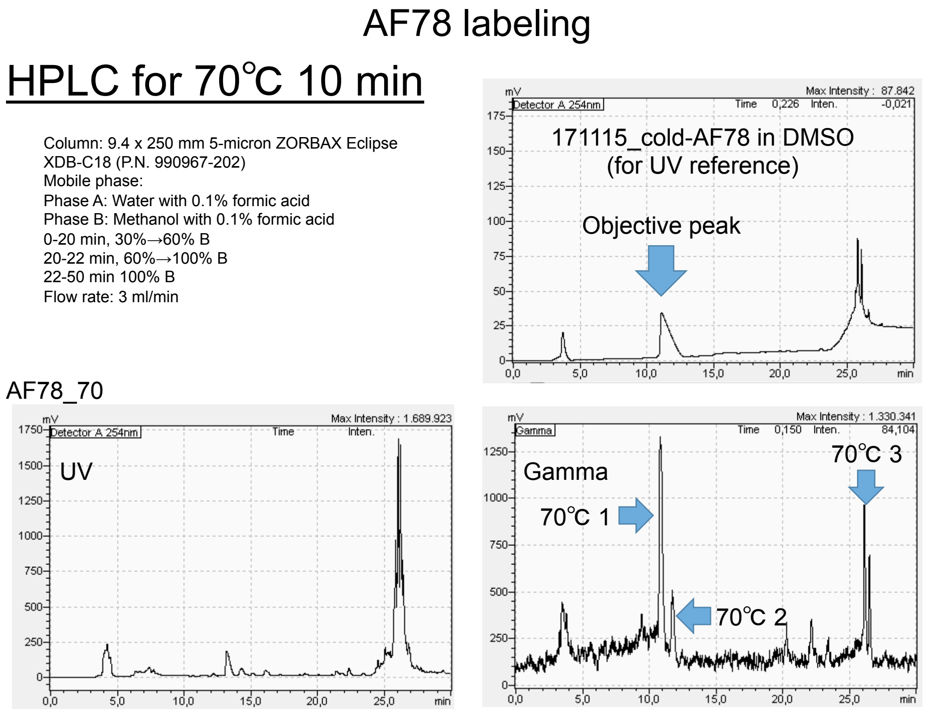

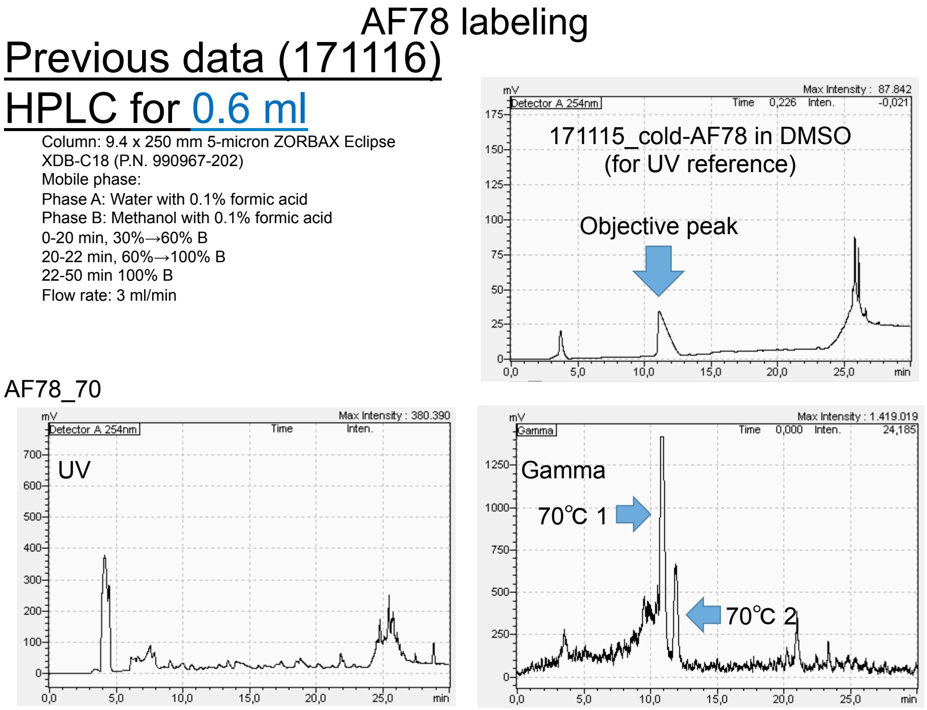

Fig. S7. HPLC with UV and gamma detector after the deprotection step in comparison with the cold reference. Conditions: above, 0.3 mL 1N HCl 70°C for 10 min; below, 0.6 mL 1N HCl 70°C for 20 min. Peak 1 is the unidentified peak that has formed, which has been confirmed still with the structure of triazanone on guanidine. Peak 2 is the target radiolabeled tracer [^{18}F]AF78.

AF78 labeling (HCl_6N_20min)

HPLC

Column: 9.4 x 250 mm 5-micron ZORBAX Eclipse XDB-C18 (P.N. 990967-202)
Mobile phase:
Phase A: Water with 0.1% formic acid
Phase B: Methanol with 0.1% formic acid
0-20 min, 30%→60% B
20-22 min, 60%→100% B
22-50 min 100% B
Flow rate: 3 ml/min

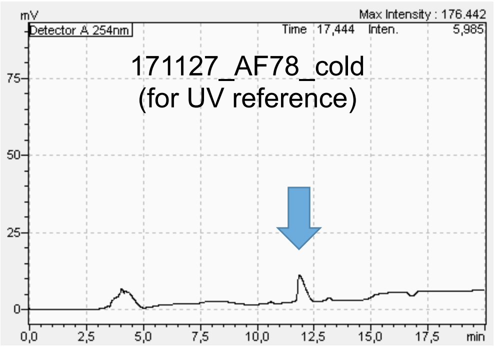

AF78

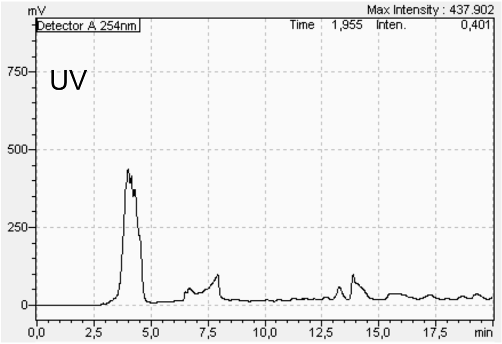

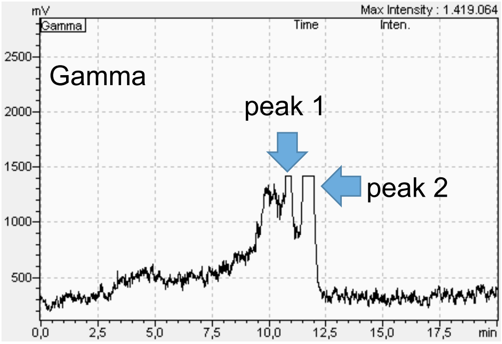

Fig. S8. HPLC with UV and gamma detector after the deprotection step in comparison with the cold reference. Conditions: 0.6 mL 6N HCl 100°C for 20 min. Peak 1 is the unidentified peak that has formed, which has been confirmed still with the structure of triazanone on guanidine. Peak 2 is the target radiolabeled tracer [^{18}F]AF78.

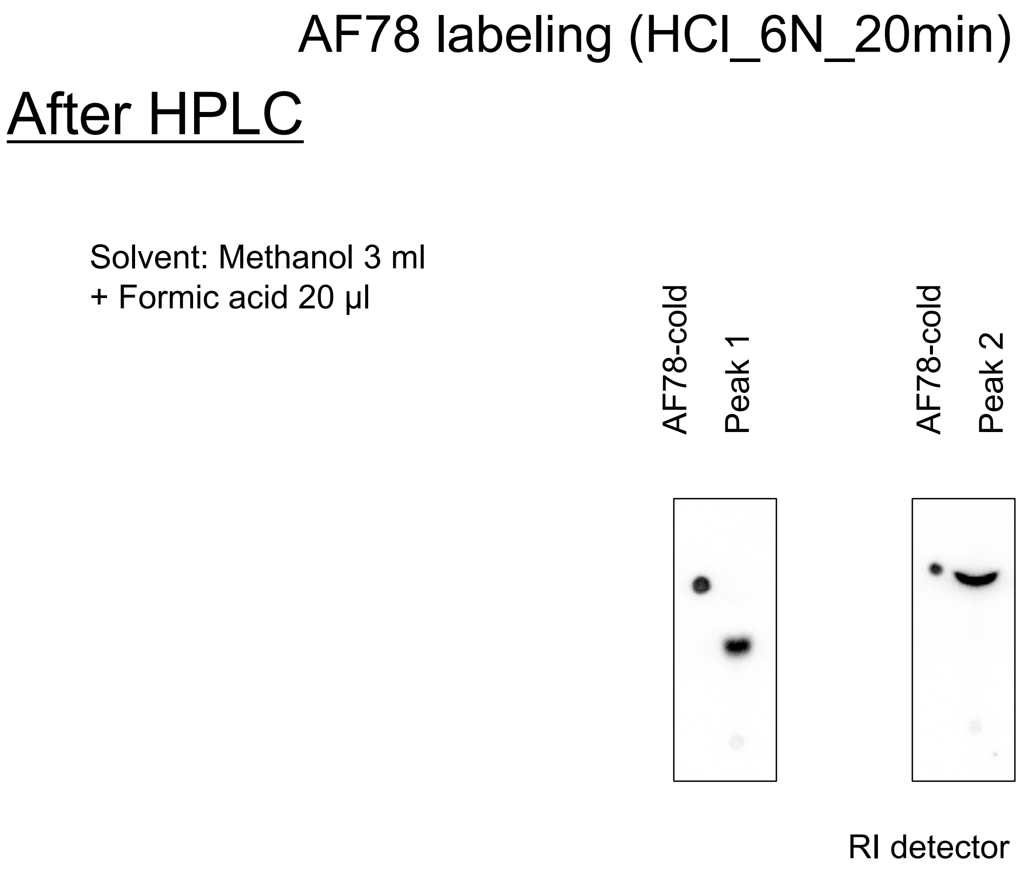

Fig. S9. Fractions after the HPLC purification as compared to the cold reference of AF78 along with the purity control using autoradiographic TLC.

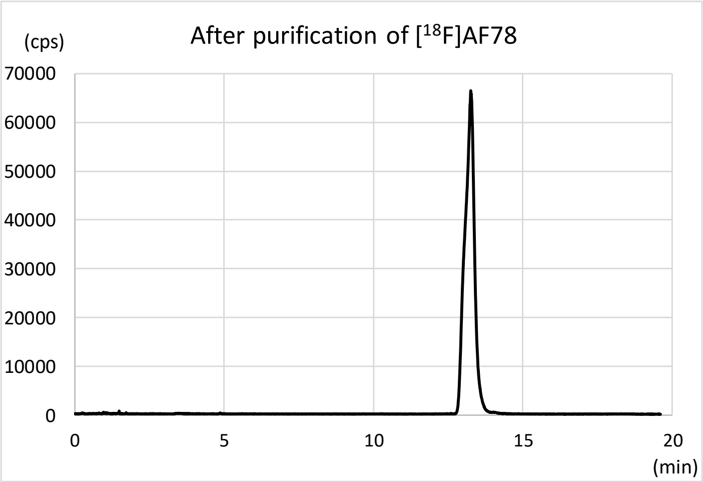

Fig. S10. HPLC with gamma detector after the purification. Column: COSMOSIL 5C18-AR-II 10ID x 250 mm (Nacalai tesque), Mobile phase: Phase A: Water with 0.1% formic acid, Phase B: Methanol with 0.1% formic acid, 0-25 min, 20%→50% B, 25-30 min, 50%→95% B, 30-35 min 98% B, Flow rate: 3 ml/min, Radiochemical purity by analytical HPLC was > 97 %.

Cell culture

Medium: for incubation 50 mL

EMEM + 2 mM glutamine + 10% FBS + P/S	50 mL
Pargyline (final 100 µM) in medium	500 µL of 100X stock
Pyrogallol (final 20 µM) in medium	50 µL of 1000X stock
Tracer in H_2O:ethanol 1:1 solution	45.2 µL (final 3.7 kBq/well)

Norepinephrine (30 mM) in medium

Cold MHPG (30 mM) in DMSO

Cold tracer 2 (30 mM) in DMSO

Desipramine (6 mM) in medium

References

S1. Ding YS, Fowler JS, Gatley SJ, Dewey SL, Wolf AP, Schlyer DJ (1991) Synthesis of high specific activity 6-[^{18}F]fluorodopamine for positron emission tomography studies of sympathetic nervous tissue. J Med Chem 34:861-863

S2. Musiol HJ, Moroder L (2001) *N,N′*-Di-*tert*-butoxycarbonyl-1*H*-benzotriazole-1-carboxamidine derivatives are highly reactive guanidinylating reagent. Org Lett 3:3859-3861

S3. Bouvet J, Cousserans G (1970) Textile finishing treatment with triazine derivatives. US patent 3,521,996

S4. Xu, RQ, Gu Q, You SL (2017) Construction of the benzomesembrine skeleton: Palladium(0)-catalyzed intermolecular acrylative dearomatization of α–naphthols and subsequent Aza-Michael reaction. Angew Chem Int Ed 56:7252-7256

S5. Watanuki S, Matsuura K, Tomura Y, et al (2012) Synthesis and pharmacological evaluation of 2-(1-alkyl-4-piperidinyl)-N-[(1R)-1-(4-fluorophenyl)-2-methylpropyl]acetamide derivatives as novel antihypertensive agents. Chem Pharm Bull 60:223-234

S6. Whitesides GM, San Filippo Jr. J (1970) Mechanism of reduction of alkylmercuric halides by metal hydrides. JACS 92:6611-6624

S7. Langer O, Dollé F, Valette H, et al (2001) Synthesis of high-specific-radioactivity 4- and 6-[^{18}F]fluorometaraminol-PET tracers for the adrenergic nervous system of the heart. Bioorg Med Chem 9:677-694

S8. Jiang JA, Chen C, Guo Y, et al (2014) A highly efficient approach to vanillin starting from 4-cresol. Green Chem 16:2807-2814
